# Podoplanin and Selected Cancer Stem Cell or Stemness-Associated Markers in Oral Epithelial Dysplasia: A Structured Narrative Review of Their Role in Malignant Transformation and Risk Stratification

**DOI:** 10.3390/reports9030316

**Published:** 2026-09-20

**Authors:** Panagiota Dimitropoulou, Filippos Fytros, Christina Charisi, Amalia Syrioti, Nikolaos Spantidakis, Konstantinos Poulopoulos, Maria Fasoula, Efstratios Karagiannidis, Athanasios Poulopoulos, Vasileios Zisis

**Affiliations:** 1 Department of Dentistry, School of Dentistry, European University Cyprus, Nicosia 2404, Cyprus; 2 Department of Oral Medicine and Pathology, School of Dentistry, Aristotle University of Thessaloniki, 54124 Thessaloniki, Greece; 3 Department of Dentoalveolar Surgery, Surgical Implantology and Radiology, School of Dentistry, Aristotle University of Thessaloniki, 54124 Thessaloniki, Greece; 4 Department of Emergency Medicine, Ahepa University Hospital, Aristotle University of Thessaloniki, 54124 Thessaloniki, Greece

**Keywords:** oral epithelial dysplasia, oral potentially malignant disorders, podoplanin, cancer stem cells, malignant transformation, risk stratification, biomarkers, oral squamous cell carcinoma

## Abstract

Background: Oral potentially malignant disorders (OPMDs) comprise clinically and histologically heterogeneous lesions associated with an increased risk of oral squamous cell carcinoma (OSCC). Histopathological grading of oral epithelial dysplasia (OED) remains central to risk estimation but has limited predictive precision. Podoplanin and selected cancer stem cell (CSC) or stemness-associated markers may capture complementary biological processes involved in progression. Objective: To synthesize evidence on podoplanin and selected CSC or stemness-associated markers as indicators of malignant transformation and risk stratification across a prespecified OPMD spectrum, while distinguishing longitudinal prediction from cross-sectional, diagnostic, and mechanistic evidence. Methods: Three original PubMed searches covered database inception to 13 July 2026. A post hoc recall audit against three key evidence syntheses identified one eligible longitudinal report missed during the initial selection; this report was added by backward citation checking. A broader supplementary sensitivity query was run during revision and reported separately without being merged into the original selection counts. In total, 47 publications were included. Study design, effect measure, threshold provenance, internal validation, and possible cohort overlap were charted and synthesized narratively. Results: Among the biomarkers reviewed, podoplanin had the most developed longitudinal evidence, including a pooled hazard ratio (HR) of 3.72 (95% confidence interval [CI] 2.40–5.76) in one meta-analysis. A separate diagnostic-accuracy synthesis reported sensitivity of 78.9% and specificity of 55.0%, corresponding to a positive likelihood ratio (LR+) of 1.75 and a negative likelihood ratio (LR−) of 0.38. Predictive values varied materially with baseline transformation risk. CD133, BMI1, and ABCG2 showed large associations in individual cohorts, but independent replication and external validation were limited. Cross-sectional expression gradients were not treated as evidence of prospective prediction. Conclusions: Within the defined OPMD scope, podoplanin has the most developed longitudinal evidence, but its predictive values are prevalence-dependent and no marker is sufficiently validated for stand-alone clinical use. Prospective multicenter studies should compare prespecified biomarker models with established clinical, histopathological, and molecular predictors and should evaluate calibration, clinical utility, and external validity before management decisions are altered.

## 1. Introduction

Oral squamous cell carcinoma (OSCC) commonly arises through a clinically recognizable precursor phase. The term oral potentially malignant disorders (OPMDs) encompasses a heterogeneous group of conditions associated with increased cancer risk, including oral leukoplakia, erythroplakia and erythroleukoplakia, proliferative verrucous leukoplakia, oral submucous fibrosis, and actinic cheilitis [1]. Oral lichen planus (OLP) and oral lichenoid lesions (OLLs) require particular diagnostic caution because epithelial dysplasia may favor reclassification as an OLL rather than OLP. Accordingly, this review did not treat all OPMDs as biologically equivalent and reports lesion-specific limitations where relevant.

Histopathological assessment of oral epithelial dysplasia (OED), based on the extent of architectural and cytological abnormalities, remains central to risk estimation. In this review, “lesion severity” refers to the clinical extent or phenotype of an OPMD and/or the histopathological grade of OED, as specified by each source; these dimensions were not assumed to be interchangeable. OED grade does not provide an absolute estimate of future behavior, and interobserver variability, sampling error, and transformation of lesions without dysplasia limit its prognostic precision [1,2].

Among biomarkers examined in oral premalignancy, podoplanin has attracted particular attention. Podoplanin is a mucin-type transmembrane glycoprotein expressed physiologically by lymphatic endothelium and may also be induced in inflammatory and reparative contexts [3]. Aberrant epithelial expression has been associated with cytoskeletal remodeling, altered adhesion, migration, and early invasion. Its expression must, therefore, be interpreted in its epithelial and tissue context rather than as a tumor-specific signal.

Cancer stem cell (CSC) and stemness-associated markers constitute a biologically related but distinct field. CD44, CD133, BMI1, CD24, NANOG, and ABCG2 have been used to identify or enrich cellular populations associated with self-renewal, persistence, therapy resistance, and tumor initiation. They are not universally accepted or interchangeable CSC identifiers; their expression is context-dependent and none alone proves a stem-cell phenotype.

Biomarker expression alone, however, does not establish prognostic validity. Cross-sectional differences among normal mucosa, dysplasia, and OSCC may support biological plausibility or diagnostic association, but they do not demonstrate that a marker can predict future malignant transformation. Direct evidence of risk stratification requires longitudinal follow-up, reliable ascertainment of transformation outcomes, and appropriate consideration of established clinical and histopathological predictors. This distinction is particularly important because many studies of podoplanin and CSC markers have focused on associations with dysplasia grade or disease stage rather than on subsequent development of OSCC [1,4].

The available literature is also methodologically heterogeneous. Studies differ in lesion definitions, patient populations, aetiological exposures, specimen types, antibodies, staining protocols, cellular localization, scoring systems, positivity thresholds, follow-up periods, and statistical analyses [1,4]. The evidence ranges from small cross-sectional immunohistochemical investigations to longitudinal cohorts, studies nested within chemoprevention trials, systematic reviews, meta-analyses, bioinformatic investigations, and single-cell transcriptomic analyses. These differences complicate direct comparison and may contribute to variation in reported associations and effect estimates.

Podoplanin was examined alongside selected CSC or stemness-associated markers because epithelial motility and invasion may interact with persistence, self-renewal, and microenvironmental signalling during malignant progression. Direct longitudinal co-evaluation of podoplanin and ALDH1 provides one clinical bridge between these domains [5]. The markers were selected because they recur in OPMD studies captured by the preliminary literature assessment and have clinical or mechanistic data in oral premalignancy; they are, therefore, a prespecified pragmatic panel, not a claim that they are the only or “principal” CSC markers. Epithelial–mesenchymal transition (EMT) was considered only as supplementary mechanistic evidence linking motility and stemness, not as an outcome equivalent to malignant transformation.

The aim of this structured narrative review was to synthesize evidence on podoplanin and selected cancer stem cell (CSC) or stemness-associated markers across the defined oral potentially malignant disorder (OPMD) spectrum; to foreground longitudinal malignant-transformation studies; to distinguish prediction from cross-sectional association and mechanistic evidence and to evaluate threshold selection, validation, cohort overlap, prevalence-dependent performance, and potential clinical utility.

## 2. Methods

### 2.1. Review Design and Scope

This study was conducted as a structured narrative review and is reported with reference to the Scale for the Assessment of Narrative Review Articles (SANRA) [6]. The structured search, evidence charting, and descriptive selection diagram were used to improve transparency; they do not convert the review into a systematic review, and the corpus should be understood as focused and illustrative rather than demonstrably exhaustive.

The primary review question concerned malignant transformation from a defined OPMD to OSCC. Longitudinal studies, therefore, received the greatest interpretive weight. Cross-sectional comparisons, recurrence studies, diagnostic-accuracy studies, and mechanistic analyses were retained only when they directly informed the prespecified biomarkers or the biological interpretation of progression. EMT was treated as a supplementary mechanism, not as a co-primary outcome.

### 2.2. Information Source and Search Strategy

PubMed was selected as the sole bibliographic database because this structured narrative review was designed as a focused synthesis of biomedical, pathology, and oral-oncology literature within a feasible review process. PubMed provides MEDLINE indexing and broad coverage of the relevant clinical and laboratory journals; however, reliance on one database reduces sensitivity compared with a multi-database systematic review and is acknowledged as a limitation. The search covered all records available to 13 July 2026.

The first search combined broad terms relating to oral epithelial dysplasia and OPMDs with terms for podoplanin and the prespecified CSC or stemness-associated markers and returned 60 records. The second search focused specifically on podoplanin in relation to oral premalignancy, oral epithelial dysplasia, and oral cancer and returned 33 records. The third search focused on CSC and stemness-associated markers in relation to oral premalignancy, oral epithelial dysplasia, and oral cancer and returned 23 records. Collectively, the three searches identified 116 records before duplicate removal.

The three PubMed search strategies were as follows:

Query 1 (broad):

(“oral epithelial dysplasia” OR “oral potentially malignant disorder*” OR OPMD OR “oral leukoplakia” OR leukoplakia) AND (podoplanin OR PDPN OR “D2-40” OR “cancer stem cell*” OR stemness OR CD44 OR NANOG OR CD133 OR BMI1) AND (“malignant transformation” OR carcinogenesis OR progression OR prognosis OR “risk stratification”)

Query 2 (podoplanin-focused):

(“oral epithelial dysplasia” OR “oral leukoplakia” OR “oral potentially malignant disorder*” OR OPMD) AND (podoplanin OR PDPN OR “D2-40”) AND (“malignant transformation” OR progression OR prognosis OR carcinogenesis)

Query 3 (cancer stem cell marker-focused):

(“oral epithelial dysplasia” OR “oral leukoplakia” OR “oral potentially malignant disorder*” OR OPMD) AND (“cancer stem cell*” OR stemness OR “stem cell marker*” OR CD44 OR CD133 OR NANOG OR BMI1 OR CD24) AND (“malignant transformation” OR progression OR prognosis OR carcinogenesis)

The three queries were deliberately complementary. Query 1 included the exact phrase “risk stratification,” whereas Queries 2 and 3 used the broader outcome concepts progression, prognosis, and carcinogenesis to avoid requiring authors to use that specific phrase. The focused queries were intended to improve precision for podoplanin and stemness literature, not to establish exhaustive recall for every OPMD subtype.

Podoplanin was searched using its standard name, gene symbol (PDPN), and the common immunohistochemical designation D2-40. Marker-specific synonyms were not exhaustively enumerated for every CSC-associated marker; instead, broad concepts such as “cancer stem cell,” “stemness,” and “stem cell marker” were combined with selected names. “Mouth Diseases” [MeSH] was not used because it is substantially broader than the lesion population of interest and would have reduced precision. Likewise, OSCC was not required as a separate outcome term because malignant transformation, progression, prognosis, and carcinogenesis were intended to retrieve progression-to-cancer studies while limiting OSCC-only literature. These choices are limitations rather than evidence of an exhaustive search.

ABCG2 and ALDH1 were not included as independent defining terms in the original queries. They were eligible when retrieved through broader stemness terminology or evaluated with a prespecified marker. Because this approach could miss reports indexed only under an individual marker name or under specific OPMDs such as actinic cheilitis, oral submucous fibrosis, or OLP, a post hoc recall audit and backward citation checking were undertaken and the residual risk of missed studies is acknowledged.

During revision, a supplementary PubMed sensitivity search was run on 3 September 2026. It explicitly enumerated the named OPMDs; marker aliases, including PROM1/AC133 (CD133), BCRP (ABCG2), and ALDH1A1 (ALDH1) and the outcome terms “risk stratification”, “oral squamous cell carcinoma”, “OSCC”, and “oral cancer”. The deliberately broad query returned 144 records. Because this post hoc search was a sensitivity check rather than a new, duplicate-screened systematic search, its records were not merged with the original source-selection counts and it was not used to claim exhaustive recall.

Supplementary sensitivity query: (“oral epithelial dysplasia” OR “oral potentially malignant disorder*” OR OPMD OR “oral leukoplakia” OR leukoplakia OR erythroplakia OR erythroleukoplakia OR “proliferative verrucous leukoplakia” OR “oral submucous fibrosis” OR “oral lichen planus” OR “oral lichenoid lesion*” OR “actinic cheilitis”) AND (podoplanin OR PDPN OR “D2-40” OR “cancer stem cell*” OR stemness OR “stem cell marker*” OR CD44 OR CD44v OR CD133 OR PROM1 OR AC133 OR NANOG OR BMI1 OR “BMI-1” OR CD24 OR ABCG2 OR BCRP OR “breast cancer resistance protein” OR ALDH1 OR ALDH1A1) AND (“malignant transformation” OR progression OR prognosis OR carcinogenesis OR “risk stratification” OR “oral squamous cell carcinoma” OR OSCC OR “oral cancer”).

### 2.3. Eligibility Criteria

Eligibility was defined operationally before synthesis. The population comprised human tissue or patient studies of oral leukoplakia, erythroplakia or erythroleukoplakia, proliferative verrucous leukoplakia, oral submucous fibrosis, actinic cheilitis, or histologically diagnosed OED. Dysplasia was not required for clinically defined OPMDs in which it is variably present. Reports labelled OLP were not pooled with other OPMDs. If epithelial dysplasia was described at baseline, the lesion was treated as an OLL or as diagnostically uncertain rather than as unequivocal OLP; progression-labelled OLP cohorts were retained only as a separate, explicitly qualified evidence stratum [7]. No unspecified residual category of “another OPMD” was used.

Studies containing both OPMD and OSCC groups were eligible only when the OPMD group was substantively and separately analyzed. Studies confined to established OSCC were excluded. OSCC comparison tissue could inform cross-sectional or mechanistic interpretation but could not, by itself, provide evidence of prediction from a premalignant state.

The prespecified core markers were podoplanin/PDPN, CD44 and its variants, CD133, BMI1, CD24, NANOG, and ABCG2. This selection reflected their recurrence in the oral-premalignancy literature and their representation of adhesion/motility, self-renewal, persistence, or drug-efflux phenotypes. The designation “core” refers only to the review scope and does not imply universal consensus that these are the principal CSC markers.

Articles were retained when at least one core marker was substantively assessed. Additional biomarkers, including ALDH1, SOX2, OCT4, ΔNp63, p75 neurotrophin receptor, and PIWIL2, were charted only when evaluated alongside a core marker or when necessary to interpret a combined model. EMT-related proteins were treated as supplementary mechanistic evidence and not placed at the same evidential level as malignant transformation or stemness.

The primary outcome was malignant transformation of an OPMD or oral epithelial dysplasia into OSCC. Secondary outcomes included time to malignant transformation, development of oral cancer during follow-up, lesion progression or recurrence, association with histopathological dysplasia grade, biomarker-based risk stratification, discrimination between lower-risk and higher-risk lesions, molecular alterations associated with oral carcinogenesis, and prognostic outcomes directly relevant to malignant progression.

Prospective and retrospective cohort studies, case-control studies, cross-sectional immunohistochemical studies, diagnostic and prognostic biomarker studies, studies nested within chemoprevention trials, systematic reviews, meta-analyses, scoping reviews, and transcriptomic or bioinformatic investigations directly relevant to OPMD progression were eligible for inclusion as evidential sources. Narrative reviews and structured commentaries were considered only when they provided relevant mechanistic or contextual interpretation and were not treated as independent primary evidence. Systematic reviews, meta-analyses, narrative reviews, and commentaries were synthesized separately from primary studies to minimise double-counting of evidence derived from overlapping cohorts.

Articles were excluded when they investigated non-oral malignancies; salivary-gland, odontogenic, or non-epithelial tumors; unrelated head-and-neck sites without a separately analyzable oral-lesion group; exclusively animal or in vitro models; or established OSCC without a separately analyzed OPMD group. The broad conceptual search terms retrieved three records in non-oral or non-epithelial settings, which were removed during title-and-abstract screening.

### 2.4. Scope Clarifications and Treatment of Additional Biomarkers

To preserve a focused review scope, studies in which ALDH1, SOX2, or OCT4 constituted the sole or principal biomarker specified in the study objective and primary analyses were excluded. Although these markers are frequently discussed within the broader stemness literature, each has an extensive and partly distinct evidence base in oral premalignancy that would require separate evaluation.

This exclusion criterion was applied at the level of the article rather than to every biomarker included within it. Studies evaluating ALDH1, SOX2, or OCT4 alongside an eligible marker, such as CD44, CD133, BMI1, NANOG, or ABCG2, were retained when the eligible marker was substantively assessed and its findings were separately reported.

During full-text assessment, the scope was further clarified to permit the inclusion of supplementary findings relating to additional biomarkers when they were evaluated alongside at least one eligible primary marker in the same study. These supplementary biomarkers included ΔNp63, p75NTR/CD271, PIWIL2, epidermal growth factor receptor, and epithelial–mesenchymal transition-associated proteins such as vimentin, E-cadherin, and Snail. These markers were not treated as primary search targets or as equivalent to the prespecified core biomarkers, but were incorporated where they provided relevant mechanistic, comparative, or multi-marker evidence.

### 2.5. Study Selection

Records retrieved through the three PubMed searches were combined and exact duplicates removed. Screening proceeded from titles and abstracts to full texts. To examine search recall, a post hoc audit compared the selected corpus with scope-eligible longitudinal primary reports represented in three key evidence syntheses: Saluja et al. [4], Monteiro et al. [8], and Briggs et al. [9]. Backward citation checking was limited to this audit and was documented separately from database retrieval.

Titles and abstracts were assessed against the eligibility criteria and classified as potentially eligible, ineligible, or requiring full-text assessment because the available information was insufficient to determine eligibility. Full texts were obtained for all potentially eligible records and for records whose eligibility could not be resolved at the title-and-abstract stage. Full-text articles were assessed using the same eligibility criteria, and reasons for exclusion were documented.

Study selection, data extraction, and critical appraisal were conducted by the first author (P.D.). The PubMed searches identified 116 records; after 53 exact duplicates were removed, 63 unique records were screened, 48 full texts were assessed, and 46 database-selected publications met the eligibility criteria. The post hoc recall audit added one eligible report [5], producing a final corpus of 47 publications.

The two systematic reviews from the same research programme [10,11] were retained because they covered sequential search periods, but their conclusions were interpreted as overlapping rather than independent evidence. The structured commentary [12] was used only for context and was not counted as original evidence.

**Figure 1:**
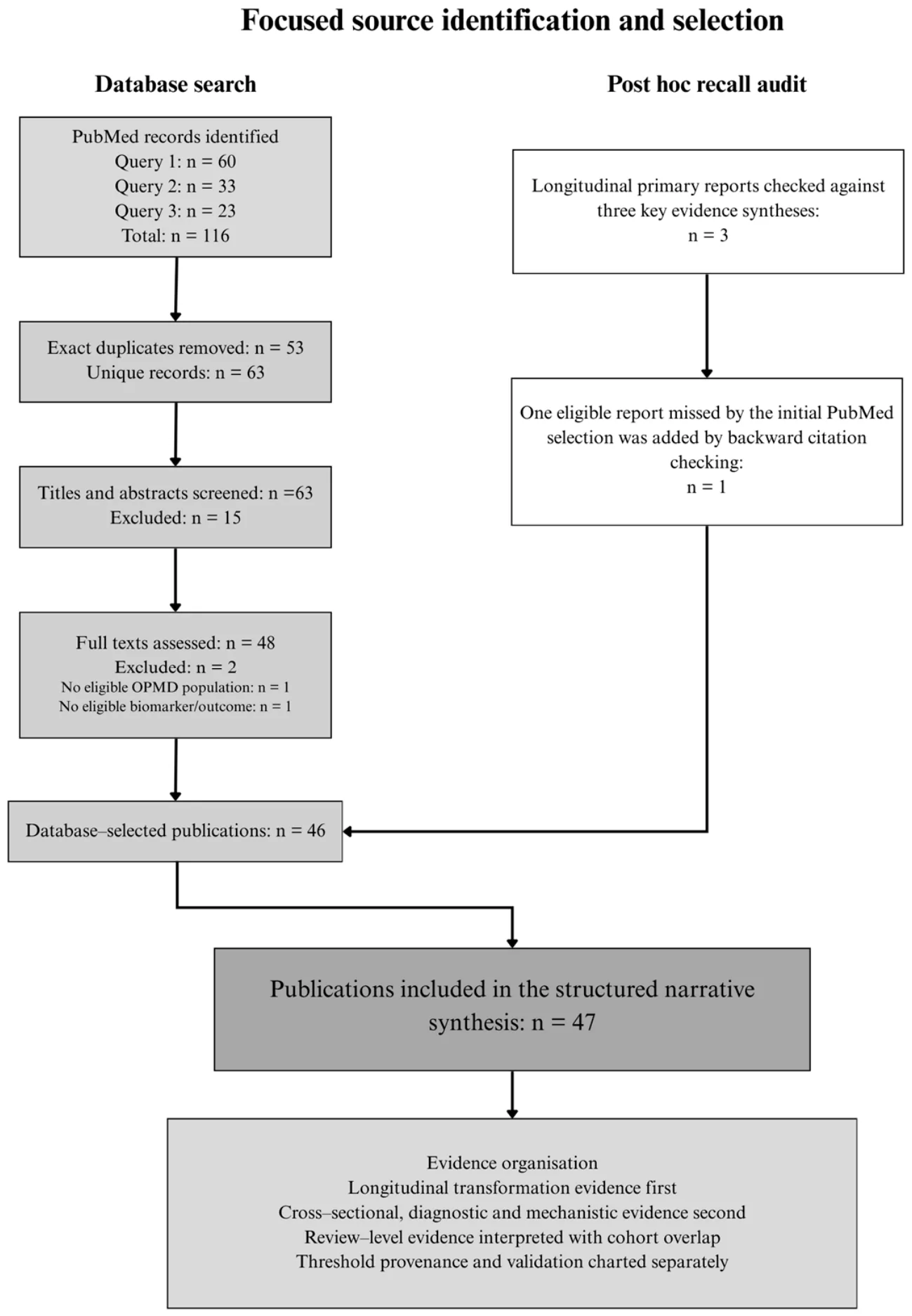
Focused source identification and selection. The post hoc recall audit was based on Saluja et al. [4], Monteiro et al. [8], and Briggs et al. [9]. Backward citation checking identified one additional eligible report by Habiba et al. [5]. This is a descriptive selection diagram for a structured narrative review and not a PRISMA flow diagram.

Figure 1 presents a descriptive source-selection diagram for this structured narrative review rather than a PRISMA flow diagram. The recall audit recovered one initially missed report, Habiba et al. [5]. After this correction, the corpus contained all scope-eligible longitudinal reports benchmarked against Saluja et al. (7/7), Monteiro et al. (6/6), and Briggs et al. (5/5), corresponding to 10/10 unique reports after overlap among the three syntheses.

### 2.6. Data Extraction and Evidence Charting

Data extraction used a structured evidence-charting framework. Extracted items included study design, setting, lesion definition, sample size, follow-up, transformation events, biomarker assay, antibody or reagent, cellular localization, scoring method, threshold, threshold provenance (prespecified, externally adopted, or data-derived), internal validation, effect measure, confidence interval (CI), adjustment variables, and potential cohort overlap. Immunohistochemistry (IHC) was recorded as the dominant tissue-assessment method.

Effect measures were retained exactly as reported and were not treated as interchangeable: hazard ratios (HRs) describe time-to-event associations, odds ratios (ORs) describe odds under the reported model, and risk ratios (RRs) compare cumulative risks. Sensitivity, specificity, positive predictive value (PPV), negative predictive value (NPV), and likelihood ratios (LRs) were recorded where available. When published sensitivity and specificity permitted, LR+ and LR− and illustrative predictive values were calculated transparently; no missing study effect estimate or CI was imputed.

In studies evaluating both prespecified eligible biomarkers and additional biomarkers, findings relating to the prespecified biomarkers were classified as core evidence. Findings concerning additional biomarkers were incorporated as supplementary evidence only where they contributed directly to the interpretation of multi-marker models, comparative biomarker performance, or biological mechanisms relevant to malignant transformation.

Detailed study characteristics, biomarker-assessment methods, scoring systems, quantitative findings, statistical adjustment, combined-model evaluation, and principal methodological limitations are presented in Supplementary Tables S1–S3.

### 2.7. Critical Appraisal of Methodological Strengths and Limitations

Because this was a structured narrative review, no protocol was registered and no numerical risk-of-bias score or pooled certainty rating was assigned. Nevertheless, each study was appraised using consistent considerations covering population definition, follow-up and event ascertainment, assay reproducibility, threshold provenance, validation, statistical model, confounding, precision, and overlap. This approach supports critical interpretation but is not equivalent to a duplicate formal risk-of-bias assessment.

For longitudinal prognostic studies, appraisal addressed the representativeness of the study population, clarity and consistency of lesion definitions, adequacy and duration of follow-up, completeness of outcome ascertainment, number of malignant-transformation events, reproducibility of biomarker measurement, prespecification of scoring thresholds, adjustment for histopathological grade and other potential confounding variables, appropriateness of the statistical analysis, and internal or external validation of predictive models.

For cross-sectional studies, appraisal focused on participant and specimen selection, appropriateness of comparison groups, standardization of biomarker assessment, blinding of histopathological or immunohistochemical evaluation, adequacy of statistical analysis, and the distinction between association with dysplasia severity and prediction of malignant transformation.

Systematic reviews and meta-analyses were considered in relation to the comprehensiveness of their search strategies, clarity of eligibility criteria, assessment of primary-study strengths and limitations, treatment of statistical and methodological heterogeneity, evaluation of publication bias, and overlap in the primary cohorts included across different reviews.

Scoping reviews, narrative reviews, and the structured commentary were evaluated with regard to the transparency of their source selection, the accuracy and balance of their interpretation, and their contribution to contextual or mechanistic understanding. These publications were not treated as independent evidence of predictive validity.

The findings of this appraisal were incorporated into the narrative interpretation of each biomarker rather than converted into a formal summary score.

### 2.8. Evidence Synthesis

A new meta-analysis was not undertaken because of substantial heterogeneity among the included studies in relation to study populations, lesion definitions, biomarker-detection methods, antibody clones, staining patterns, scoring systems, positivity thresholds, follow-up durations, and reported effect measures.

The evidence was synthesized narratively and organized primarily according to biomarker. Within each biomarker category, studies were interpreted according to the nature of the evidence provided. Longitudinal studies reporting malignant transformation were distinguished from cross-sectional studies examining dysplasia grade or lesion severity, diagnostic studies evaluating discriminatory performance, mechanistic or transcriptomic investigations, and studies evaluating multi-marker or biomarker-plus-histology models.

Evidence was synthesized narratively by biomarker and ordered within each subsection by evidential relevance: longitudinal malignant-transformation studies first, followed by cross-sectional, diagnostic, or mechanistic evidence. When no eligible longitudinal transformation study existed for a marker, this was stated explicitly.

Systematic reviews and meta-analyses were synthesized separately from primary studies. Overlap in the primary studies included across different reviews was considered in order to avoid interpreting repeated analyses of the same cohorts as fully independent replication.

Where findings differed between studies, potential explanations were examined, including variation in lesion type, aetiological exposure, follow-up duration, sample size, antibody protocol, cellular localization, scoring system, biomarker threshold, and adjustment for histopathological grade or other confounding variables.

## 3. Results

### 3.1. Study Identification and Characteristics

The three PubMed queries identified 116 records. After removal of 53 exact duplicates, 63 unique records were screened; 15 were excluded at the title and abstract stage and 48 full texts were assessed. Two full texts were excluded, leaving 46 database-selected publications. The post hoc recall audit added Habiba et al. [5] through backward citation checking, yielding 47 publications in the final narrative synthesis (Figure 1).

The final corpus comprised nine review-level or contextual publications [1,2,3,4,8,9,10,11,12] and 38 original investigations [5,13,14,15,16,17,18,19,20,21,22,23,24,25,26,27,28,29,30,31,32,33,34,35,36,37,38,39,40,41,42,43,44,45,46,47,48,49]. Fourteen original studies provided direct longitudinal or retrospectively reconstructed malignant-transformation evidence [5,15,16,17,18,20,25,26,36,37,38,39,41,46]; one assessed recurrence without transformation as its primary outcome [21], one focused mainly on survival across OED and OSCC [40], 20 were cross-sectional or diagnostic studies, including one transformed-versus-non-transformed comparison without a time-to-event analysis [13,14,19,22,23,24,27,28,29,30,31,33,34,35,42,43,44,45,47,48], and two were transcriptomic or bioinformatic analyses [32,49]. References [6,7,50,51,52,53] are methodological or contextual citations added during revision and are not part of this 47-publication evidence corpus.

The review-level evidence included a scoping review [1], narrative or structured contextual reviews [3,12], systematic reviews [10,11], and meta-analyses focused on podoplanin or prognostic biomarkers [2,4,8,9]. All syntheses were examined for overlap in their underlying cohorts.

IHC was used as a primary or confirmatory method in 36 of the 38 original investigations; the principal exceptions were a salivary molecular study [31] and a transcriptomic meta-analysis [49]. D2-40 was the most frequently named podoplanin antibody, although incomplete reporting prevented a defensible exact count across studies. Oral leukoplakia was represented in at least 25 original investigations and was the most common clinical diagnosis; other populations included histologically defined OED, erythroplakia, OLP/OLL, oral submucous fibrosis, proliferative verrucous leukoplakia, and actinic cheilitis (Supplementary Tables S1 and S2).

Follow-up in longitudinal studies ranged from less than 2 years to more than 20 years, and malignant transformation was the principal longitudinal outcome [5,15,16,17,18,20,25,26,36,37,38,39,41,46]. Cross-sectional differences in expression by lesion category or dysplasia grade, including a descriptive transformed-versus-non-transformed comparison [43], were treated as biological or diagnostic associations and not as equivalent to prospective prediction [13,14,19,22,23,24,27,28,29,30,31,33,34,35,42,43,44,45,47,48].

### 3.2. Podoplanin as a Biomarker of Malignant Transformation

Longitudinal evidence for podoplanin came from prospective or retrospective OPMD cohorts [5,15,16,17,18,19,20,25,26]. Across these studies, positive epithelial expression was generally associated with a higher transformation rate, although effect size, statistical significance, and independence from histological grade varied with the scoring rule and analytical model. Cross-sectional expression gradients are considered after the longitudinal findings.

Kawaguchi et al. [15] followed 150 patients with oral leukoplakia for a median of 7.5 years. Podoplanin positivity remained associated with oral cancer development in multivariable Cox regression (HR 3.09, 95% CI 1.53–6.23). Three-year cancer incidence ranged from 3% in lesions negative for both dysplasia and podoplanin to 38% in lesions positive for both, supporting joint interpretation with histopathology rather than stand-alone testing.

De Vicente et al. [16] reported an adjusted HR of 8.738 (95% CI 1.834–41.634) in 58 dysplastic oral leukoplakias. Monteiro et al. [17] reported an HR of 10.148 (95% CI 1.503–68.532) for their study-specific podoplanin score and an HR of 10.238 (95% CI 2.060–50.889) when podoplanin was combined with dysplasia grade. These large but imprecise estimates had wide CIs, and only the study-specific score—not the externally applied layer-based score—remained significant in the latter cohort, underscoring sensitivity to threshold and scoring choices.

Kreppel et al. [18] observed five-year oral cancer-free survival of 100% among podoplanin-negative patients and 41.7% among those with the highest score, but podoplanin did not remain significant after multivariable adjustment. A later prospective study from the group linked podoplanin to dysplasia grade rather than directly establishing transformation prediction [19]. Habiba et al. [5] reported adjusted HRs of 2.62 (95% CI 1.04–7.59) for podoplanin, 3.02 (95% CI 1.39–7.38) for ALDH1, and 3.64 (95% CI 1.71–8.25) for their co-expression in 79 oral leukoplakias; the study used a minimum six-month latency rule but had a short mean follow-up of 3.4 years.

At review level, Saluja et al. [4] reported a pooled RR of 3.45 for malignant progression in podoplanin-positive lesions, and Mello et al. [1] found positive associations in all eight podoplanin studies they identified. Monteiro et al. [8], pooling six cohorts [5,15,16,17,18,19], estimated an HR of 3.72 (95% CI 2.40–5.76) with I^2^ = 0%. Turton et al. [2] also identified podoplanin, together with epidermal growth factor receptor expression, p16 methylation, and DNA aneuploidy, among biomarkers with statistically significant pooled longitudinal associations. I^2^ = 0% should not be read as methodological uniformity, because the small number of studies limits heterogeneity detection and the cohorts differed in scoring and thresholds.

Briggs et al. [9] reported a pooled HR of 4.4 (95% CI 2.6–7.4), sensitivity of 78.9%, and specificity of 55.0%. These estimates correspond to LR+ = 1.75 and LR− = 0.38. The published PPV of 36.4% and NPV of 88.8% reflect the event prevalence in the contributing data and are not transportable constants. As Table 1 illustrates, NPV remains high at lower baseline risks while the post-test risk after a negative result increases as baseline risk rises; podoplanin should, therefore, not be used alone to reduce surveillance.

Longitudinal findings were not uniformly positive. Verma and Chandrashekar [20] found an association with dysplasia grade but not subsequent transformation in 60 OED cases. Sundberg et al. [21] observed a non-significant tendency toward higher expression in recurrent leukoplakia, but recurrence was not equivalent to malignant transformation.

Cross-sectional studies commonly reported increasing epithelial podoplanin expression with dysplasia or OSCC [13,14,23,24], but this pattern was not uniform. Alkan et al. [22] found no significant difference among proliferative verrucous leukoplakia, conventional leukoplakia, and OLP despite transformation rates of 77.1%, 2.5%, and 2.1%, respectively. D’Souza et al. [23] likewise found no significant overall difference between leukoplakia and healthy tissue. These results show that expression gradients cannot substitute for longitudinal discrimination.

In the ten-biomarker prognostic analysis by Zhang et al. [25], podoplanin was significant in univariable analysis but was not retained in the final model, which combined p53 and carbonic anhydrase 9 with age and dysplasia grade. By contrast, Saintigny et al. [26] reported an adjusted HR of 3.308 (95% CI 1.663–6.580) for ΔNp63 and an HR of 4.372 (95% CI 1.912–9.992) for a combined ΔNp63–podoplanin–inflammatory-cell model. These findings support integrated modeling but remain cohort-specific.

### 3.3. CD44 and CD44 Variants

As a prespecified predictor of malignant transformation. The available evidence was principally cross-sectional or diagnostic. Kuo et al. [27] found high expression of several CD44 isoforms across normal, hyperkeratotic, and dysplastic epithelium, whereas loss of CD44v7–8 emerged mainly in OSCC. Dhumal et al. [28] likewise reported a non-linear pattern. These data do not support a uniform stage-wise increase or establish prospective prognostic validity.

Surendran et al. [29] reported increasing CD44 expression with dysplasia severity and co-expression with CD31, CXCR4, and stromal cell-derived factor 1 in higher-risk lesions. The study supports a vascular-niche hypothesis but did not provide longitudinal transformation estimates for CD44.

The strongest clinically oriented CD44 evidence came from diagnostic rather than prognostic work. Sunny et al. [30] evaluated a CD44/SNA-1 cytology assay across three cohorts, reporting sensitivity above 83% for separating low-risk lesions from high-grade dysplasia or carcinoma and above 88% after integration with clinical variables and machine learning. These results support triage research, not replacement of biopsy or prediction of future transformation.

Shah et al. [31] detected salivary CD44 variants and reported associations with disease severity. Choi et al. [32] identified fibroblast-derived COL1A1–CD44 signalling at the leukoplakia stage and in carcinoma in situ cell populations not recognized on routine histopathology. The latter finding is mechanistically important but also raises the possibility that a biomarker may reveal already-present, unsampled neoplasia rather than predict a new future event.

Additional cross-sectional studies reported CD44 expression across OED and OSCC [33,34,35], but small dysplasia subgroups, heterogeneous thresholds, and absent longitudinal follow-up limit risk-prediction inferences. Collectively, CD44 has supportive biological and diagnostic evidence but insufficient direct evidence for malignant-transformation prediction.

### 3.4. CD133, BMI1, and ABCG2

Longitudinal evidence was available for CD133, BMI1, and ABCG2, usually from single-center retrospective cohorts [36,37,38,41]. ALDH1 findings are reported only where that marker was co-evaluated with an eligible core marker [5,37,39], consistent with the prespecified scope.

Liu et al. [36] reported adjusted ORs—not time-to-event HRs—of 3.24 (95% CI 1.31–7.98) for ABCG2 and 4.03 (95% CI 1.59–10.26) for BMI1 in 135 patients with oral leukoplakia. A later report from the same institution [37] used survival analysis and reported HRs of 4.17 (95% CI 1.96–8.90) for ALDH1 and 2.86 (95% CI 1.48–5.55) for CD133 in 141 patients. Similar recruitment setting and follow-up characteristics mean that patient overlap cannot be excluded; the reports should not automatically be counted as fully independent cohorts.

Sun et al. [38] reported an OR of 9.79 (95% CI 1.96–48.92) for CD133 positivity in progression-labelled OLP. The estimate was based on only 10 progression events and was imprecise. Diagnostic uncertainty between OLP, OLL, and dysplastic lichenoid lesions further limits generalization.

Saluja et al. [4] reported pooled RRs of 4.20 for CD133 and 3.64 for ABCG2. These RRs should not be conflated with the cohort ORs or HRs, and the small numbers of eligible studies and potential Shanghai-cohort overlap reduce the apparent independence of the evidence.

Feng et al. [39] described ALDH1/BMI1 co-expression in oral erythroplakia followed by multiple or multifocal carcinomas, but the brief report did not provide an HR, OR, or CI and may overlap with other Shanghai cohorts [36,37,38]. Rao et al. [40] contributed cross-sectional and survival-related evidence across OED and OSCC rather than direct OPMD transformation estimates.

Tarle et al. [41] reported an HR of 12.84 (95% CI 2.15–76.44) for ABCG2/nuclear epidermal growth factor receptor co-expression. The very wide CI, small sample (*n* = 50), and weaker independent performance of ABCG2 alone indicate that the estimate is imprecise and primarily supports investigation of a combined model.

Not all findings were favorable. Klein and colleagues found that BMI1 expression was significantly higher in leukoplakia than in normal oral mucosa but did not differ significantly between dysplastic and non-dysplastic leukoplakia. This finding limits its potential value for risk stratification within an existing OPMD population, despite its ability to distinguish abnormal from normal tissue [42].

### 3.5. Supplementary Biomarkers Evaluated Alongside Eligible Markers

Longitudinal supplementary evidence concerned ΔNp63, PIWIL2, and selected co-marker models [26,46]. Matsubara et al. [43] provided a retrospective transformed-versus-non-transformed comparison without a time-to-event estimate. Cross-sectional EMT and microenvironmental studies were considered only after these reports and were not treated as outcomes equivalent to malignant transformation.

Saintigny et al. [26] reported an adjusted HR of 3.308 (95% CI 1.663–6.580) for ΔNp63 and an HR of 4.372 (95% CI 1.912–9.992) for the combined ΔNp63–podoplanin–inflammatory-cell model. Matsubara et al. [43] observed higher ΔNp63 labelling in transformed than non-transformed oral leukoplakia, but only six transformations occurred and no multivariable time-to-event estimate was reported.

No eligible longitudinal malignant-transformation study was identified for p75 neurotrophin receptor/CD271. Cross-sectional studies by Kiyosue et al. [44] and Abdulmajeed et al. [45] did not show reliable discrimination among OED grades, limiting their relevance to premalignant risk stratification.

Wang et al. [46] identified PIWIL2 as an independent predictor in a retrospective oral-leukoplakia cohort; CD133 and NANOG were tissue comparators rather than separately powered longitudinal predictors. Attrition from 208 reviewed to 101 analyzable cases and incomplete threshold reporting limit interpretation.

Cross-sectional EMT studies supplied supplementary mechanistic evidence. Miguel et al. [47] found an association between discontinuous podoplanin expression and reduced E-cadherin, consistent with—but not proof of—a link to EMT. Tokozlu et al. [48] detected Snail upregulation in many dysplastic and hyperplastic specimens; frequent expression in non-dysplastic hyperplasia limited specificity.

The transcriptomic findings of Vasudevan et al. [49] supported, rather than independently established, the conclusion that stemness-associated pathways are involved in OPMD progression: a stemness-induction network was enriched in transformed lesions. The study did not validate an individual protein marker or estimate patient-level predictive performance.

### 3.6. Systematic Reviews, Meta-Analyses, and Evidence Overlap

Review-level publications synthesized overlapping primary evidence and were, therefore, used to contextualize, not multiply, independent support [1,2,3,4,8,9,10,11,12]. The structured commentary [12] provided no original data, and the transcriptomic meta-analysis [49] addressed pathway-level rather than clinical biomarker performance.

Among the selected markers, podoplanin had the most developed longitudinal and meta-analytic evidence [1,2,4,8,9]. This statement is restricted to the review scope. CD44, CD133, BMI1, and ABCG2 had less independent longitudinal replication and external validation, and none was among the four biomarkers with statistically significant pooled longitudinal associations in Turton et al. [2].

The podoplanin meta-analysis by Monteiro et al. [8] pooled six cohorts [5,15,16,17,18,19], several of which were also evaluated individually here. The pooled and individual reports were, therefore, interpreted with explicit attention to shared primary evidence, not as independent replications.

### 3.7. Methodological Heterogeneity Across the Included Literature

Methodological heterogeneity involved lesion definitions, recruitment, follow-up, antibody clones and dilutions, staining localization, scoring rules, threshold provenance, and adjustment. Supplementary Table S2 now states whether each longitudinal threshold was prespecified, externally adopted, or data-derived and whether internal validation was reported.

Podoplanin thresholds variously used epithelial-layer extent, staining intensity, percentage positivity, or composite scores [5,15,16,17,18,19]. In Monteiro et al. [17], the data-derived study score remained significant whereas an externally adopted layer-based rule did not. Very large HRs from small, same-cohort models should, therefore, be interpreted in light of cut-off selection, wide CIs, and absent resampling or external validation rather than as necessarily stronger biological effects.

Follow-up ranged from less than 2 years to more than 20 years, and studies used different latency rules. Liu et al. [37] and Habiba et al. [5] required at least six months between index diagnosis and carcinoma, whereas other reports did not state an equivalent rule. Short intervals may capture carcinoma already present but unsampled at baseline, while long follow-up increases the opportunity to observe a genuinely subsequent event. Choi et al. [32] directly illustrated occult carcinoma in situ cell populations missed by routine histopathology.

Study populations differed in lesion composition and predominant exposures. Betel-quid-associated cohorts and tobacco- or alcohol-associated cohorts may not be biologically exchangeable, yet exposure was inconsistently incorporated into adjusted analyses [27,29,36,37,38]. In addition, overlapping recruitment at the Shanghai institution across Liu et al., Sun et al., and Feng et al. could not be excluded [36,37,38,39], reducing the effective number of independent replications.

OLP/OLL findings were interpreted separately because diagnostic criteria and the handling of epithelial dysplasia vary. A lichenoid lesion with dysplasia may be classified as OLL rather than OLP, and studies based on progression-labelled OLP may include heterogeneous entities [7,38]. These data should not be pooled uncritically with oral leukoplakia or OED cohorts.

Principal quantitative findings, adjustment variables, combined- or multi-marker model evaluations, and the main methodological limitations of the included publications are summarized in Supplementary Table S3.

## 4. Discussion

This structured narrative review organizes 47 publications into an evidence hierarchy that separates longitudinal prediction from cross-sectional association, documents a post hoc recall audit, distinguishes HRs, ORs, and RRs, evaluates threshold provenance and validation, and accounts for possible cohort overlap. Its contribution is, therefore, critical synthesis rather than a new pooled effect estimate.

Within the selected marker set, podoplanin has the most developed longitudinal evidence [1,2,4,5,8,9,15,16,17,18,19,20]. The pooled sensitivity and specificity reported by Briggs et al. [9] correspond to LR+ = 1.75 and LR− = 0.38, indicating modest rule-in and more useful—but incomplete—rule-out information. The reported NPV of 88.8% is prevalence-dependent and does not justify reduced surveillance without validation in the intended clinical population.

CD133, BMI1, and ABCG2 showed comparatively large ORs, HRs, or pooled RRs [4,36,37,38,41], but these effect measures answer different questions and should not be interchanged. The small number of cohorts, wide CIs, and possible overlap among Shanghai reports [36,37,38,39] leave their independent reproducibility uncertain.

No individual marker is ready for stand-alone clinical risk stratification. Combined biomarker–histology or multivariable models sometimes improved discrimination [5,15,17,25,26,41], but most thresholds were study-defined or data-derived, internal validation was uncommon, and external validation was generally absent.

Biologically, podoplanin and stemness-associated markers may capture connected but non-equivalent processes. Podoplanin can influence cytoskeletal organization, adhesion, and motility, whereas CSC-associated markers may reflect persistence or self-renewal [3,26,29,51]. Podoplanin is also a lymphatic endothelial marker and can be induced in inflammatory settings [51]; epithelial localization and microenvironmental context are, therefore, essential to interpretation.

Large associations for CD133, BMI1, and ABCG2 are biologically plausible [36,37,38,41], but marker positivity does not itself establish a functional CSC population. The discordance of podoplanin and ΔNp63 in approximately one-third of lesions [26] supports partial complementarity rather than a single shared pathway.

Mechanistic studies support a model of epithelial–stromal interaction but do not prove prospective prediction. Reduced E-cadherin with discontinuous podoplanin expression [47], vascular-niche co-expression around CD44 [29], and COL1A1–CD44 signalling [32] are consistent with early motility and stemness interactions. The transcriptomic analysis [49] supports involvement of stemness-associated networks; it does not independently validate that conclusion at the protein or patient level. Detection of carcinoma in situ cell populations by Choi et al. [32] also suggests that some apparently predictive signals may reflect occult disease already present at baseline.

Predictive values must be interpreted at the intended baseline risk. Using Briggs et al.’s pooled sensitivity and specificity [9], the illustrative PPV increases from 8.4% at a 5% baseline risk to 36.9% at 25%, while the NPV decreases from 98.0% to 88.7% (Table 1). These calculations clarify why neither a positive nor a negative podoplanin result should independently determine excision, surveillance intensity, or reassurance.

Integrated models combining clinical variables, OED grade, and molecular markers are a plausible research direction [5,15,17,25,26,41]. However, evidence that biomarker-guided excision prevents cancer is lacking, and available intervention evidence does not establish an effective chemopreventive strategy for oral leukoplakia [50]. Prospective studies should evaluate an explicit management strategy—test, threshold, resulting action, benefits, and harms—rather than biomarker association alone.

PDPN should also be benchmarked directly against established or more broadly validated molecular approaches, including DNA ploidy/aneuploidy, loss-of-heterozygosity profiles, and pattern-based p53/p16 IHC [1,2,52,53]. The present conclusion that podoplanin is comparatively mature applies only among the markers reviewed here; it is not a ranking across the entire OPMD biomarker field. Future models should report discrimination, calibration, decision-curve or other clinical-utility measures, incremental value, and external validation.

Most primary studies were single- or dual-center, retrospective, and based on modest event counts, leading to imprecise estimates and unstable multivariable models [5,16,17,18,20,41,43]. Thresholds were often study-defined or data-derived, and few reports used bootstrap, cross-validation, split-sample validation, or an external cohort (Supplementary Table S2).

Publication bias and selective reporting remain plausible, particularly in small biomarker studies. Moreover, I^2^ = 0% in a six-study meta-analysis [8] does not erase clear clinical and methodological heterogeneity; with few studies, heterogeneity tests have low power, and selective reporting can further narrow the observed distribution of effects.

Assay heterogeneity included antibody clone and dilution, epithelial versus stromal localization, scoring components, and positivity thresholds [5,15,16,17,18,19]. The contrast between study-specific and externally adopted podoplanin scores within the same cohort [17] illustrates how analytical choices can influence statistical significance and apparently large HRs.

Follow-up and outcome definitions varied, including whether a minimum latency separated the index biopsy from carcinoma [5,37]. Logistic ORs from variable follow-up cohorts [36,38] are not equivalent to Cox HRs [5,15,16,17,37,41] because only the latter explicitly incorporate event timing. Potential overlap among Shanghai cohorts [36,37,38,39] further reduces the effective independence of the evidence.

Null findings are biologically informative. Podoplanin failed to distinguish lesion categories with markedly different observed transformation rates in Alkan et al. [22], and BMI1 did not separate dysplastic from non-dysplastic leukoplakia in Klein et al. [42]. Because podoplanin may be induced in inflammatory or reparative contexts and is physiologically expressed in lymphatic endothelium [51], variable inflammatory composition and cellular localization may partly contribute to discordant results.

This review has important limitations. It is a single-reviewer, PubMed-only structured narrative review without a registered protocol, duplicate screening or extraction, formal risk-of-bias scoring, or a new meta-analysis. The focused original queries did not enumerate every synonym, marker, MeSH heading, or individual OPMD; the post hoc recall audit improved detection of known longitudinal cohorts but cannot prove exhaustive recall. The broad supplementary sensitivity search was not integrated into a duplicate-screened systematic update. English-language and indexing restrictions, incomplete reporting, unresolved cohort overlap, and reliance on published aggregate data may, therefore, have affected the corpus and interpretation.

## 5. Conclusions

Across the explicitly defined OPMD spectrum, podoplanin has the most developed longitudinal evidence among the markers reviewed, but LR+ = 1.75 and LR− = 0.38 indicate only moderate diagnostic information [9]. PPV and NPV vary with baseline risk, and neither positivity nor negativity is sufficiently validated to determine surveillance or treatment independently.

CD44 has mainly cross-sectional and diagnostic evidence, whereas CD133, BMI1, and ABCG2 have longitudinal signals from a small and potentially overlapping set of cohorts. The current evidence supports investigation of integrated clinical–histopathological–molecular models rather than reliance on any single marker.

Future research should prioritise prospective multicenter cohorts with explicit baseline sampling, latency definitions, blinded and standardized assays, prespecified thresholds, adequate event numbers, internal resampling, and independent external validation. Studies should benchmark new models against OED grade, DNA ploidy, p53-pattern, and loss-of-heterozygosity approaches and should evaluate a complete clinical strategy—including the action triggered by a result, benefits, harms, calibration, and net clinical utility—before implementation.

## Figures and Tables

**Table 1:** Illustrative podoplanin predictive values across plausible baseline malignant-transformation risks.

Baseline Risk	PPV	NPV	Risk After Negative Result
5%	8.4%	98.0%	2.0%
10%	16.3%	95.9%	4.1%
15%	23.6%	93.7%	6.3%
20%	30.5%	91.2%	8.8%
25%	36.9%	88.7%	11.3%

Note: Values are calculated from the pooled sensitivity (78.9%) and specificity (55.0%) reported by Briggs et al. [9], assuming these estimates remain constant across baseline risks. They are illustrative and are not externally validated management thresholds. PPV, positive predictive value; NPV, negative predictive value.

## Data Availability

The original data presented in the study are included in the article; further inquiries can be directed to the corresponding author.

## Supplementary Materials

The following supporting information can be downloaded at: https://www.mdpi.com/article/10.3390/reports9030316/s1, Table S1. Study design, population characteristics, follow-up, and outcomes of the included publications. Publications are ordered according to the main manuscript reference list. Follow-up and transformation or progression events are reported only where applicable; review-level, cross-sectional, diagnostic, mechanistic, and transcriptomic publications are identified explicitly. Table S2. Biomarker-assessment methods, scoring systems, and positivity or analytical thresholds. For primary studies, assay type, antibody or reagent details, quantification method, and cut-offs are reported where available. For review-level publications, the table summarises heterogeneity in the methods and thresholds used by the underlying studies. Table S3. Principal findings, statistical adjustment, combined-model evaluation, and methodological limitations. Effect estimates and confidence intervals are reproduced as reported. Combined-model status distinguishes formal multivariable or multi-marker models from descriptive co-expression or mechanistic analyses.
