# Supplementary material for: Podoplanin and Selected Cancer Stem Cell or Stemness-Associated Markers in Oral Epithelial Dysplasia: A Structured Narrative Review of Their Role in Malignant Transformation and Risk Stratification
Source: Reports (MDPI). 2026 Sep 20;9(3):316. doi: 10.3390/reports9030316 (PMC13611620; doi:10.3390/reports9030316)
Supplement: Supplementary file 1 [file reports-09-00316-s001.zip › reports-4487925-supplementary.pdf]

Supplementary Material

Supplementary Tables S1–S3 each contain 47 study rows corresponding to main-manuscript references [1–5,8–49]. Reference [5] was added after the post hoc recall audit. The sequential Monteiro reviews [10,11] are retained as distinct publications but interpreted as overlapping evidence. Data are reproduced as reported by the source publications. NR means not reported; “not reported in sufficient detail” indicates that the report did not permit a more precise extraction; and an em dash indicates that a field was not applicable. No unreported effect estimate, confidence interval, or P value was imputed. Supplementary Table S2 additionally records threshold provenance and internal validation for longitudinal studies.

Table S1. Study design, population characteristics, follow-up, and outcomes of the included publications. Publications are ordered according to the main manuscript reference list. Follow-up and transformation or progression events are reported only where applicable; review-level, cross-sectional, diagnostic, mechanistic, and transcriptomic publications are identified explicitly.

Ref.	Study	Evidence Category	Design and Country/ Setting	Lesion or Population and Sample Size	Follow-up and Transformation/Progression Events
**[1]**	Mello et al., 2020	Review-level (scoping review)	Scoping review; multi-country evidence synthesis	OPMDs (various subtypes)<br>73 longitudinal studies, 5612 OPMD cases, 108 biomarkers	Aggregate across 73 studies; individual follow-up and event counts varied by primary study.
**[2]**	Turton et al., 2024	Review-level (systematic review/meta-analysis)	Systematic review and meta-analysis restricted to longitudinal studies; multi-country evidence synthesis	OED<br>20 studies (of 2829 identified), 32 biomarkers	Longitudinal design required for inclusion<br>Aggregate, not individually reported
**[3]**	Swain et al., 2014	Review-level (narrative/mechanistic)	Narrative mechanistic review	No primary population	—
**[4]**	Saluja et al., 2019	Review-level (meta-analysis)	Meta-analysis of primary studies; multi-country evidence synthesis	OPMDs (leukoplakia, erythroplakia, OLP, others)<br>12 primary studies / 18 investigations, ~1659 patients pooled	Pooled across included studies; individual follow-up and event counts were not separately reported.
[5]	Habiba et al., 2017	Longitudinal (retrospective cohort)	Retrospective follow-up cohort; Japan (Hokkaido University Hospital)	Oral leukoplakia, n = 79 (27 low-grade and 52 high-grade dysplasia)	Mean 3.4 years; 37 transformations (46.8%); minimum six-month latency.
**[8]**	Monteiro et al., 2024	Review-level (systematic review/meta-analysis)	Systematic review and meta-analysis; PROSPERO CRD42022329326	Oral leukoplakia<br>6 studies, 546 patients	32–90 months<br>125 cancers (22.9%)

Ref.	Study	Evidence Category	Design and Country/ Setting	Lesion or Population and Sample Size	Follow-up and Transformation/Progression Events
**[9]**	Briggs et al., 2025	Review-level (systematic review/meta-analysis)	Systematic review and meta-analysis; multi-country evidence synthesis	OPMD, OSCC, healthy controls (14 pooled studies)<br>*N* = 971 (78.7% OPMD, 13.9% OSCC, 7.4% controls)	Pooled transformation data were available from five included studies; aggregate follow-up was not reported in sufficient detail.
**[10]**	Monteiro et al., 2021	Review-level (systematic review)	Systematic review; multi-country evidence synthesis	Oral leukoplakia<br>46 studies, 3783 patients	Aggregate across 46 studies; 1047 malignant transformations (27.6%).
**[11]**	Monteiro et al., 2025	Review-level (systematic review)	Prospectively registered systematic review; multi-country evidence synthesis	Oral leukoplakia<br>14 studies (of 1385 identified), 1479 patients	Aggregate across 14 studies; 216 malignant transformations (14.6%).
**[12]**	Cousins & Carney, 2022	Review-level (commentary)	Structured commentary on Monteiro et al. [10]	No independent population; discusses the 46 studies and 3783 patients synthesised in Monteiro et al. [10].	—
**[13]**	Logeswari et al., 2014	Cross-sectional	Retrospective cross-sectional<br>NR	Normal gingiva; oral leukoplakia; OSCC<br>*n* = 10 normal, 32 leukoplakia, 50 OSCC	No longitudinal follow-up (cross-sectional study).
**[14]**	Gissi et al., 2018	Cross-sectional / diagnostic	Retrospective/cross-sectional<br>NR	Oral leukoplakia, dysplastic versus non-dysplastic<br>*n* = 8 dysplastic, 32 non-dysplastic	No longitudinal follow-up (cross-sectional/diagnostic study).
**[15]**	Kawaguchi et al., 2008	Longitudinal (prospective cohort)	Prospective cohort nested in RCT<br>USA (MD Anderson Cancer Center)	Oral leukoplakia<br>*n* = 150	Median 7.5 years<br>35 cancers (23%)
**[16]**	de Vicente et al., 2013	Longitudinal (retrospective cohort)	Retrospective cohort<br>Spain (Oviedo)	Oral epithelial dysplasia<br>*n* = 58	Minimum 5 years or until progression<br>13 transformations (22.4%)
**[17]**	Monteiro et al., 2022	Longitudinal (retrospective cohort)	Retrospective cohort<br>NR	Oral leukoplakia (41 low-grade, 11 high-risk dysplasia) + normal controls<br>*n* = 52 OL + 12 controls	Malignant-transformation-free survival analysed; event count and follow-up duration were not reported in sufficient detail.
**[18]**	Kreppel et al., 2012	Longitudinal (retrospective cohort)	Retrospective cohort<br>Germany (Cologne)	Oral leukoplakia<br>*n* = 60	Structured recall at 3–6-month intervals; exact transformation-event count was not reported; 5-year oral cancer-free survival was reported.

Ref.	Study	Evidence Category	Design and Country/ Setting	Lesion or Population and Sample Size	Follow-up and Transformation/Progression Events
**[19]**	Grochau et al., 2019	Cross-sectional association (prospective recruitment)	Prospective observational study with cross-sectional biomarker–dysplasia-grade analysis; Germany (Cologne)	Oral leukoplakia (consecutive, treatment-naive) *n* = 50	No longitudinal transformation or survival analysis reported.
**[20]**	Verma & Chandrashekar, 2019	Longitudinal (cohort with transformation outcome, underpowered)	Retrospective cross-sectional/cohort NR	Oral epithelial dysplasia (20 mild, 20 moderate, 20 severe/CIS) *n* = 60	Follow-up duration not reported in sufficient detail; 15/60 lesions (25%) transformed.
**[21]**	Sundberg et al., 2021	Longitudinal (prospective cohort)	Prospective study NR	Oral leukoplakia (33 recurrent, 40 non-recurrent) *n* = 73	Median 3.1 years overall (1.4 years to recurrence) 33 recurrences
**[22]**	Alkan et al., 2022	Cross-sectional (with retrospective clinical-outcome comparison)	Retrospective clinico-pathological comparative study NR	PVL, leukoplakia without dysplasia, OLP/OLL, verrucous carcinoma *n* = 134 (35 PVL, 40 LK, 48 OLP/OLL, 11 VC)	Retrospective clinical history; follow-up duration not reported in sufficient detail. Transformation rates: PVL 77.1%, leukoplakia 2.5%, and OLP/OLL 2.1%.
**[23]**	D'Souza et al., 2018	Cross-sectional	Retrospective/cross-sectional NR	Homogeneous OL, non-homogeneous OL, healthy controls n = 15 per group	No longitudinal follow-up (cross-sectional study).
**[24]**	Shabir et al., 2024	Cross-sectional	Retrospective analytical cross-sectional Pakistan (Peshawar)	Benign, dysplastic, OSCC *n* = 68 (20 benign, 20 dysplastic, 28 OSCC)	No longitudinal follow-up (cross-sectional study).
**[25]**	Zhang et al., 2017	Longitudinal (retrospective cohort, prediction-model development)	Retrospective cohort (nomogram development) South Korea	Oral leukoplakia *n* = 160 (22 transformed, 138 untransformed)	Median 11.3 years (range 4.6–23.2) 22 transformations (median 3.3 years to conversion)
**[26]**	Saintigny et al., 2009	Longitudinal (prospective cohort)	Prospective cohort nested in RCT USA (MD Anderson)	Oral leukoplakia *n* = 152	Median 7.5 years 36 cancers (24%)
**[27]**	Kuo et al., 1998	Cross-sectional (with secondary OSCC survival analysis)	Cross-sectional IHC comparison Taiwan	OSCC, epithelial dysplasia (ED), epithelial hyperkeratosis (EH), normal buccal mucosa *n* = 62 OSCC, 10 ED, 10 EH, 10 NBM	Five-year survival analysis in the OSCC group only; no malignant-transformation follow-up for the premalignant groups.

Ref.	Study	Evidence Category	Design and Country/ Setting	Lesion or Population and Sample Size	Follow-up and Transformation/Progression Events
**[28]**	Dhumal et al., 2022	Cross-sectional	Cross-sectional comparative study<br>NR	Normal, OPMD (OL + OSMF, dysplastic/non-dysplastic), OSCC<br>*n* = 25 normal, 30 OPMD (22 dysplastic: 15 high-risk/7 low-risk; 8 non-dysplastic), 24 OSCC	No longitudinal follow-up (cross-sectional study).
**[29]**	Surendran et al., 2017	Cross-sectional	Retrospective cross-sectional<br>NR	Non-dysplastic, mild/moderate/severe dysplasia, carcinoma, normal<br>550 samples / 349 patients	No longitudinal follow-up (cross-sectional study).
**[30]**	Sunny et al., 2023	Diagnostic (cross-sectional validation)	Multi-stage diagnostic-accuracy study (discovery + IHC + cytology + ML validation)<br>NR	Benign/hyperplasia/low-risk lesions through high-grade dysplasia/cancer<br>IHC *n* = 131; cytology *n* = 133; ML *n* = 138	No longitudinal follow-up (diagnostic-classification study).
**[31]**	Shah et al., 2018	Cross-sectional / diagnostic (biofluid)	Bioinformatic discovery + cross-sectional biofluid validation<br>NR	OSCC, oral leukoplakia, controls<br>*n* = 30 OSCC, 10 leukoplakia + controls (saliva/serum)	No longitudinal follow-up (cross-sectional biofluid study).
**[32]**	Choi et al., 2023	Transcriptomic / mechanistic	Single-cell RNA-seq + bulk-cohort correlation + in-vitro functional validation<br>South Korea (Ajou University)	Normal, leukoplakia, primary carcinoma, and lymph-node metastasis; 23 patients contributing samples across four tissue categories (normal *n* = 9, leukoplakia *n* = 4, primary carcinoma *n* = 20, lymph-node metastasis *n* = 4); 54,239 cells.	Cross-sectional single-cell profiling; no longitudinal follow-up.
**[33]**	He et al., 2014	Cross-sectional (CD44 secondary; OSCC survival analysis)	Tissue-microarray IHC with retrospective survival follow-up restricted to the OSCC subgroup; China (Wuhan)	Normal, OED, OSCC<br>*n* = 16–17 normal, 6–7 OED, 43 OSCC	Follow-up of 11–40 months was available only for the OSCC subgroup (*n* = 38 analysable); the OED subgroup was not followed for malignant transformation.
**[34]**	Momin et al., 2023	Cross-sectional (descriptive)	Cross-sectional descriptive<br>Pakistan (Karachi)	Dysplasia, OSCC<br>*n* = 95 (41 dysplasia, 54 OSCC)	No longitudinal follow-up (descriptive cross-sectional study).

Ref.	Study	Evidence Category	Design and Country/ Setting	Lesion or Population and Sample Size	Follow-up and Transformation/Progression Events
**[35]**	Onofrei et al., 2025	Cross-sectional	Retrospective cross-sectional<br>Romania (Iasi)	OLK, OLP, actinic cheilitis, OSCC, normal<br>n = 145 (82 OPMD/malignant, 63 normal)	No longitudinal follow-up (cross-sectional study).
**[36]**	Liu et al., 2012	Longitudinal (retrospective cohort)	Retrospective cohort<br>China (Shanghai)	Oral leukoplakia<br>n = 135	Mean 5.5 years<br>32 transformations (23.7%)
**[37]**	Liu et al., 2013	Longitudinal (retrospective cohort)	Retrospective cohort from the same Shanghai institution as Liu et al. [36]; patient independence could not be confirmed from the available recruitment details.	Oral leukoplakia<br>n = 141	Mean 5.5 years<br>37 transformations (26.2%, minimum 6-month latency rule)
**[38]**	Sun et al., 2013	Retrospective case-control study based on longitudinal progression status	Retrospective case-control study based on longitudinal progression status; China (Shanghai)	Oral lichen planus (progressing versus non-progressing) + normal + OSCC-from-OLP<br>n = 110 OLP (100 non-progressing, 10 progressing) + 10 normal + 6 archival OSCC	Mean 56 months<br>10 progression events
**[39]**	Feng et al., 2020	Longitudinal (re-analysis of retrospective cohort)	Brief re-analysis from the same Shanghai research group as Liu et al. [36,37] and Sun et al. [38]; partial patient overlap cannot be excluded.	Oral erythroplakia; sample size not reported in sufficient detail in the brief report (re-analysis of a previously reported cohort).	Follow-up duration not reported in sufficient detail; outcome was subsequent development of multiple or multifocal carcinomas.
**[40]**	Rao et al., 2020	Longitudinal (retrospective cohort, outcome primarily OSCC survival)	Retrospective cohort with survival follow-up<br>NR	OED, OSCC<br>n = 40 OED + 40 OSCC	Follow-up of 6–52 months; event count not reported in sufficient detail; survival analysis centred on the OSCC subgroup.
**[41]**	Tarle et al., 2022	Longitudinal (retrospective cohort)	Retrospective cohort<br>NR	OPMD (oral leukoplakia + oral erythroplakia)<br>n = 50 (31 OL, 19 OE)	Follow-up duration not reported in sufficient detail; 21/50 patients (42%) developed OSCC.

Ref.	Study	Evidence Category	Design and Country/ Setting	Lesion or Population and Sample Size	Follow-up and Transformation/Progression Events
**[42]**	Klein et al., 2020	Cross-sectional	Cross-sectional observational<br>NR	Non-dysplastic OL, dysplastic OL, normal, inflammatory hyperplasia, OSCC<br>n = 28 non-dysplastic OL, 33 dysplastic OL, 9 normal, 17 IH, 19 OSCC	No defined longitudinal transformation cohort; associations were based on retrospective clinical history.
**[43]**	Matsubara et al., 2011	Cross-sectional (with descriptive transformed-versus-non-transformed comparison)	Retrospective cross-sectional<br>Japan (Kyushu University)	Oral leukoplakia (67.9% hyperplasia to 5.4% severe dysplasia), OSCC, normal<br>n = 112 OL, 81 OSCC, 10 normal	Follow-up duration and the denominator for the non-transformed group were not reported in sufficient detail; 6/112 oral leukoplakias (5.4%) transformed.
**[44]**	Kiyosue et al., 2013	Cross-sectional (null finding for premalignant group)	Retrospective cross-sectional study; related Kyushu University cohort to Matsubara et al. [43]; Japan	Oral leukoplakia, OSCC<br>n = 112 OL, 81 OSCC	No malignant-transformation follow-up reported for the oral leukoplakia group.
**[45]**	Abdulmajeed et al., 2013	Diagnostic (two-stage training/validation)	Two-stage diagnostic-accuracy (training/validation) cross-sectional study<br>Australia (Queensland)	Normal, mild dysplasia, moderate/severe dysplasia, OSCC<br>Training n = 107; Validation n = 278	No longitudinal follow-up (diagnostic-classification study).
**[46]**	Wang et al., 2019	Longitudinal (retrospective cohort) plus mechanistic in vitro component	Retrospective cohort + cross-sectional OSCC cohort + in vitro<br>China	Oral leukoplakia, OSCC<br>101 OL (of 208 reviewed) + 137 OSCC (separate cohort)	Patients enrolled from 2005 to 2016; 27/101 oral leukoplakias transformed.
**[47]**	Miguel et al., 2023	Cross-sectional	Retrospective cross-sectional<br>NR	Leukoplakia with epithelial dysplasia (38 low-risk, 23 high-risk)<br>n = 61	No longitudinal follow-up (histological risk-grade study).
**[48]**	Tokozlu et al., 2024	Cross-sectional (protein + transcript level)	Retrospective comparative study<br>Turkey	OED, OSCC, and oral epithelial hyperplasia; sample size not reported in sufficient detail by group.	No longitudinal follow-up.
**[49]**	Vasudevan et al., 2026	Transcriptomic (bioinformatic meta-analysis)	Bioinformatic meta-analysis of five public transcriptomic datasets	Low-risk lesions, low-grade dysplasia, high-grade dysplasia, and transformed lesions; aggregate sample sizes were not reported in sufficient detail.	Cross-sectional transcriptomic synthesis; no patient-level longitudinal follow-up.

Table S2. Biomarker-assessment methods, scoring systems, and positivity or analytical thresholds. For primary studies, assay type, antibody or reagent details, quantification method, and cut-offs are reported where available. For review-level publications, the table summarises heterogeneity in the methods and thresholds used by the underlying studies.

Ref.	Study	Biomarker(s) and Assessment Method	Scoring or Quantification Method and Positivity/ Analytical Threshold	Threshold Provenance	Internal Validation
**[1]**	Mello et al., 2020	A total of 108 biomarkers, including podoplanin, p53, Ki-67, p16, and DNA ploidy. Across the underlying studies, 72 of 108 biomarkers were assessed by IHC.	No standardised scoring system across the included studies; thresholds varied by primary study.	Varied by source or not applicable.	Not applicable to a new patient-level prediction model.
**[2]**	Turton et al., 2024	32 biomarkers incl. podoplanin, EGFR, p16 methylation, DNA aneuploidy<br>Method: Pooled IHC/molecular data	Scoring systems and analytical thresholds varied by biomarker and primary study.	Varied by source or not applicable.	Not applicable to a new patient-level prediction model.
**[3]**	Swain et al., 2014	Podoplanin. Narrative synthesis of prior IHC studies; no new laboratory assay.	—	Varied by source or not applicable.	Not applicable to a new patient-level prediction model.
**[4]**	Saluja et al., 2019	CD133, ABCG2, podoplanin, and other CSC-related markers. Pooled IHC data from the underlying studies.	Scoring systems and positivity cut-offs varied by primary study.	Varied by source or not applicable.	Not applicable to a new patient-level prediction model.
[5]	Habiba et al., 2017	Podoplanin and ALDH1 / IHC; D2-40 (1:100, Vector Laboratories) and anti-ALDH1 clone 44/ALDH1 (1:100, BD Biosciences)	No staining or basal-only staining was negative; extension into suprabasal epithelial layers was positive.	Biologically defined epithelial-layer rule stated in the report; independent derivation was not described.	No bootstrap, cross-validation, split-sample, or external validation reported.
**[8]**	Monteiro et al., 2024	Podoplanin<br>Method: Pooled IHC (5 cohort studies + 1 RCT)	Scoring systems and positivity cut-offs varied among the six primary studies.	Varied by source or not applicable.	Not applicable to a new patient-level prediction model.
**[9]**	Briggs et al., 2025	Podoplanin<br>Method: Pooled IHC data across included studies	Scoring systems and positivity cut-offs varied across the pooled primary studies.	Varied by source or not applicable.	Not applicable to a new patient-level prediction model.
**[10]**	Monteiro et al., 2021	Forty-nine tissue biomarkers, including podoplanin, p53, Ki-67, p16, DNA ploidy, cytokeratins, and immune-cell markers (CD68, CD86, and CD163). Heterogeneous IHC and molecular methods across primary studies.	No single standardised scoring system; cut-offs varied by primary study.	Varied by source or not applicable.	Not applicable to a new patient-level prediction model.

Ref.	Study	Biomarker(s) and Assessment Method	Scoring or Quantification Method and Positivity/ Analytical Threshold	Threshold Provenance	Internal Validation
**[11]**	Monteiro et al., 2025	Thirty tissue biomarkers, most frequently BMI1, CD3/CD8, Ki-67, p16, and DNA ploidy. Heterogeneous IHC and molecular methods across primary studies.	No single standardised scoring system; cut-offs varied by primary study.	Varied by source or not applicable.	Not applicable to a new patient-level prediction model.
**[12]**	Cousins & Carney, 2022	No independent biomarker assessment; structured commentary on Monteiro et al. [10].	—	Varied by source or not applicable.	Not applicable to a new patient-level prediction model.
**[13]**	Logeswari et al., 2014	Podoplanin<br>Method: IHC (antibody clone NR)	Presence/absence by diagnostic group; positivity cut-off not reported.	Study-defined or not reported; no prespecification claim was made.	Not applicable to longitudinal prediction or not reported.
**[14]**	Gissi et al., 2018	Podoplanin, p53, Ki-67<br>Method: IHC	Presence/absence or study-specific semiquantitative assessment; positivity cut-off not reported in sufficient detail.	Study-defined or not reported; no prespecification claim was made.	Not applicable to longitudinal prediction or not reported.
**[15]**	Kawaguchi et al., 2008	Podoplanin<br>Method: IHC, D2-40 (1:100)	0–4 epithelial layer-extent score. Positivity cut-off: score ≥2.	Prespecified epithelial-layer cut-off (score ≥2) before outcome modelling.	No bootstrap, cross-validation, or external validation reported.
**[16]**	de Vicente et al., 2013	Podoplanin<br>Method: IHC, D2-40 (1:100)	Kawaguchi-derived epithelial layer-extent scoring system. Positivity cut-off: score ≥2.	Externally adopted from Kawaguchi et al. [15] (score ≥2).	No internal resampling or external validation reported.
**[17]**	Monteiro et al., 2022	Podoplanin, CD44v6, CD147, EGFR, p53, p63, p73, p16<br>Method: IHC	Study-specific combined extent-and-intensity podoplanin score; the Kawaguchi layer-extent score was also applied for direct comparison. Positivity cut-off: study-specific for the authors' score and score ≥2 for the Kawaguchi system.	Study-specific score and cut-off were derived in the same cohort; the Kawaguchi rule was an external comparator.	No bootstrap, cross-validation, split-sample, or external validation reported.
**[18]**	Kreppel et al., 2012	Podoplanin<br>Method: IHC (clone NR)	Scoring system and positivity cut-off not reported in sufficient detail.	Not reported in sufficient detail.	No internal or external validation reported.

Ref.	Study	Biomarker(s) and Assessment Method	Scoring or Quantification Method and Positivity/ Analytical Threshold	Threshold Provenance	Internal Validation
**[19]**	Grochau et al., 2019	Podoplanin Method: IHC	Extent of epithelial podoplanin expression assessed in relation to squamous intraepithelial neoplasia classification. Positivity cut-off not reported in sufficient detail.	Study-defined cross-sectional scoring rule.	Not applicable to longitudinal prediction.
**[20]**	Verma & Chandrashekar, 2019	SOX2, podoplanin Method: IHC	SOX2 H-score (0–291) and podoplanin visual score (0–3). Positivity cut-offs not reported in sufficient detail.	Study-defined; derivation not reported in sufficient detail.	No internal or external validation reported.
**[21]**	Sundberg et al., 2021	p53, p63, Ki-67, podoplanin Method: IHC (image-analysis quantification; podoplanin scored visually)	Percentage positivity for p53, p63, and Ki-67; visual scoring for podoplanin. Podoplanin cut-off not reported in sufficient detail.	Study-defined recurrence analysis; no malignant-transformation threshold.	No internal or external validation reported.
**[22]**	Alkan et al., 2022	Podoplanin, p53, COX-2 Method: IHC histomorphometric analysis	Histomorphometric IHC assessment; scoring system and positivity cut-off not reported in sufficient detail.	Study-defined or not reported; no pre-specification claim was made.	Not applicable to longitudinal prediction or not reported.
**[23]**	D'Souza et al., 2018	Podoplanin Method: IHC, D2-40	Epithelial extent and intensity scoring, with separate assessment of lymphatic vessel density. Positivity cut-off not reported in sufficient detail.	Study-defined or not reported; no pre-specification claim was made.	Not applicable to longitudinal prediction or not reported.
**[24]**	Shabir et al., 2024	Podoplanin Method: IHC	Pathologist-reviewed IHC categories; scoring system and positivity cut-off not reported in sufficient detail.	Study-defined or not reported; no pre-specification claim was made.	Not applicable to longitudinal prediction or not reported.
**[25]**	Zhang et al., 2017	p53, Ki-67, p16, beta-catenin, c-jun, c-met, IMP-3, COX-2, podoplanin, CA9 (10-marker panel) Method: IHC	Marker-specific IHC scoring systems were applied before prediction-model development; individual positivity cut-offs were not reported in sufficient detail.	Marker handling and model selection were data-driven in the development cohort.	Repeated simulation/internal model-selection assessment; no external validation.

Ref.	Study	Biomarker(s) and Assessment Method	Scoring or Quantification Method and Positivity/ Analytical Threshold	Threshold Provenance	Internal Validation
**[26]**	Saintigny et al., 2009	DeltaNp63, intraepithelial inflammatory cell clusters (EIC), podoplanin (reused from Kawaguchi cohort)<br>Method: IHC, monoclonal antibody 4A4 (1:400) for DeltaNp63	ΔNp63 composite score of 0–9 and EIC presence/absence. Positivity cut-offs: ΔNp63 score 6–9; EIC defined as ≥10 contiguous intraepithelial inflammatory cells.	ΔNp63 threshold adopted from prior work; podoplanin rule externally adopted; inflammatory-cell rule study-defined.	No resampling or external validation reported.
**[27]**	Kuo et al., 1998	CD44s, CD44v5, CD44v6, CD44v7-8<br>Method: IHC, 4 monoclonal antibodies (2C5, VFF-8, VFF-18, VFF-17)	Semiquantitative membrane-staining categories: none, <10%, 11–50%, and 51–100% positive cells.	Study-defined or not reported; no pre-specification claim was made.	Not applicable to longitudinal prediction or not reported.
**[28]**	Dhumal et al., 2022	CD44, ALDH1<br>Method: IHC	Staining index; low- and high-risk dysplasia classified using Kujan criteria. Biomarker positivity cut-offs not reported in sufficient detail.	Study-defined or not reported; no pre-specification claim was made.	Not applicable to longitudinal prediction or not reported.
**[29]**	Surendran et al., 2017	CD44, CD31, CXCR4, SDF-1<br>Method: IHC (CD44 cross-validated with 2 antibody clones)	High/low dichotomisation for each marker; numerical cut-offs not reported in sufficient detail.	Study-defined or not reported; no pre-specification claim was made.	Not applicable to longitudinal prediction or not reported.
**[30]**	Sunny et al., 2023	CD44, Cyclin D1, SNA-1, MAA (IHC panel); CD44+SNA-1 (cytology panel)<br>Method: IHC + oral cytology + machine learning	Study-specific or model-derived classification cut-offs were used across the IHC, oral-cytology, and machine-learning stages; exact cut-offs differed across stages.	Assay-stage thresholds were developed across study cohorts.	Multi-cohort diagnostic evaluation; no longitudinal prognostic validation.
**[31]**	Shah et al., 2018	CD44v6, CD44v10, SYNE1, miR34a<br>Method: Real-time PCR (ddCt) + methylation-specific PCR, salivary/serum samples	Relative mRNA quantification using the $2^{-\Delta\Delta Ct}$ method; analytical cut-off not reported in sufficient detail.	Study-defined or not reported; no pre-specification claim was made.	Not applicable to longitudinal prediction or not reported.
**[32]**	Choi et al., 2023	CD44 (with COL1A1 ligand-receptor partner)<br>Method: scRNA-seq, IHC spatial confirmation, siRNA knockdown functional assays	No traditional IHC score or positivity cut-off; CD44 was identified through transcriptomic, spatial, and functional analyses.	Study-defined or not reported; no pre-specification claim was made.	Not applicable to longitudinal prediction or not reported.

Ref.	Study	Biomarker(s) and Assessment Method	Scoring or Quantification Method and Positivity/Analytical Threshold	Threshold Provenance	Internal Validation
**[33]**	He et al., 2014	CD68, CD163 (TAM markers), SOX2, ALDH1, CD44 (CSC markers)<br>Method: IHC, Aperio digital histoscore quantification	Continuous intensity-weighted Aperio histoscore; no fixed positivity cut-off.	Study-defined or not reported; no pre-specification claim was made.	Not applicable to longitudinal prediction or not reported.
**[34]**	Momin et al., 2023	CD44<br>Method: IHC, rabbit monoclonal (1:100)	Membranous positivity reported as frequency/percentage; positivity cut-off not reported.	Study-defined or not reported; no pre-specification claim was made.	Not applicable to longitudinal prediction or not reported.
**[35]**	Onofrei et al., 2025	DEC1, CD44<br>Method: IHC (intensity + extent scoring)	Ordinal intensity and extent scores. Positivity cut-off not reported in sufficient detail.	Study-defined or not reported; no pre-specification claim was made.	Not applicable to longitudinal prediction or not reported.
**[36]**	Liu et al., 2012	ABCG2, BMI-1<br>Method: IHC, anti-ABCG2 (1:100, Santa Cruz), anti-BMI-1 (1:150, Abcam)	ABCG2 membranous/cytoplasmic and BMI1 nuclear expression classified according to previously published criteria. Exact numerical cut-offs were not reported in sufficient detail.	Previously published marker criteria; exact numerical cut-offs not reported.	No internal or external validation reported.
**[37]**	Liu et al., 2013	ALDH1, CD133<br>Method: IHC, anti-ALDH1A1 (1:250, Abcam), anti-CD133/1 (1:50, Miltenyi)	Positivity defined as staining in >5% of epithelial cells.	Study-defined >5% positivity threshold.	No internal or external validation reported.
**[38]**	Sun et al., 2013	CD133<br>Method: IHC, anti-CD133 (1:50, Miltenyi)	Positivity defined as staining in ≥5% of epithelial cells.	≥5% threshold adopted from the related laboratory protocol.	No internal or external validation reported.
**[39]**	Feng et al., 2020	ALDH1, Bmi1<br>Method: IHC	Scoring system and positivity cut-offs not reported in sufficient detail.	Not reported in sufficient detail.	No internal or external validation reported.
**[40]**	Rao et al., 2020	ALDH1, Bmi1, OCT4<br>Method: IHC	Scoring system and positivity cut-offs not reported in sufficient detail.	Study-defined or not reported; no pre-specification claim was made.	Not applicable to longitudinal prediction or not reported.

Ref.	Study	Biomarker(s) and Assessment Method	Scoring or Quantification Method and Positivity/ Analytical Threshold	Threshold Provenance	Internal Validation
**[41]**	Tarle et al., 2022	Nuclear EGFR (nEGFR), ABCG2<br>Method: IHC	Scoring system and positivity cut-offs not reported in sufficient detail.	Not reported in sufficient detail.	No internal or external validation reported.
**[42]**	Klein et al., 2020	Ki-67, BMI-1<br>Method: IHC	Marker-specific IHC quantification; BMI1 positivity cut-off not reported in sufficient detail.	Study-defined or not reported; no pre-specification claim was made.	Not applicable to longitudinal prediction or not reported.
**[43]**	Matsubara et al., 2011	DeltaNp63 (keratinocyte stem cell marker), Ki-67, CK14<br>Method: IHC, clone 4A4 (1:200)	Continuous ΔNp63 labelling index used for oral leukoplakia analyses; OSCC cases were dichotomised at the mean labelling index of 73.8% for survival analysis. No premalignant positivity cut-off was applied.	Continuous labelling index for OPMD analysis; no transformation cut-off.	No longitudinal internal or external validation.
**[44]**	Kiyosue et al., 2013	p75NTR (CD271), Ki-67, CK5, CK14<br>Method: IHC	Continuous p75NTR labelling index used for oral leukoplakia analyses; a 53.1% cut-off was applied only to the OSCC survival analysis.	Study-defined or not reported; no pre-specification claim was made.	Not applicable to longitudinal prediction or not reported.
**[45]**	Abdulmajeed et al., 2013	ALDH1a1, CD271/p75NTR, CD44, CD24<br>Method: IHC (4 markers)	Composite score of 0–12 (intensity 0–3 multiplied by percentage-positive category 0–4); training-set thresholds were applied blindly in the validation set.	Training-set thresholds were applied blindly to a validation set.	Internal split-sample validation for diagnostic classification; not longitudinal.
**[46]**	Wang et al., 2019	PIWIL2 (+ CD133, NANOG, Notch1 as comparators)<br>Method: IHC, polyclonal PIWIL2 (1:200, Abcam), integral optical density quantification	Integral optical-density quantification; positivity cut-off not reported in sufficient detail.	Cut-off derivation not reported in sufficient detail.	No internal or external validation reported.
**[47]**	Miguel et al., 2023	Vimentin, E-cadherin (EMT), podoplanin, osteopontin, IL-6, p53, Ki-67<br>Method: IHC	Marker-specific IHC expression patterns and localisation were assessed; positivity cut-offs were not reported in sufficient detail.	Study-defined or not reported; no pre-specification claim was made.	Not applicable to longitudinal prediction or not reported.
**[48]**	Tokozlu et al., 2024	Snail (EMT TF), CD133 (CSC); CD44, E-cadherin<br>Method: IHC + RT-qPCR (Snail, CD133); CD44/E-cadherin by IHC only	IHC and RT-qPCR quantification; scoring systems and positivity or analytical cut-offs were not reported in sufficient detail.	Study-defined or not reported; no pre-specification claim was made.	Not applicable to longitudinal prediction or not reported.

Ref.	Study	Biomarker(s) and Assessment Method	Scoring or Quantification Method and Positivity/ Analytical Threshold	Threshold Provenance	Internal Validation
**[49]**	Vasudevan et al., 2026	No single named protein marker (pathway/hub-gene level)<br>Method: WGCNA + differential gene expression analysis; digital cytometric deconvolution	WGCNA threshold: $p < 0.05$ and ∣correlation coefficient∣ ≥0.30; differential-expression threshold: $p < 0.05$ and fold change ≥1.5.	Varied by source or not applicable.	Not applicable to a new patient-level prediction model.

Table S3. Principal findings, statistical adjustment, combined-model evaluation, and methodological limitations. Effect estimates and confidence intervals are reproduced as reported. Combined-model status distinguishes formal multivariable or multi-marker models from descriptive co-expression or mechanistic analyses.

Ref.	Study	Principal Finding (95% CI Where Reported)	Adjustment Variables	Combined or Multi-Marker Model	Principal Methodological Limitation
**[1]**	Mello et al., 2020	8/8 podoplanin studies positive for transformation; 5/5 DNA-ploidy studies positive; no pooled effect size/CI computed	—	No	No pooled effect estimate or formal certainty-of-evidence grading; substantial overlap with primary studies included and appraised separately in the present review.
**[2]**	Turton et al., 2024	4/32 biomarkers meta-analytically significant: podoplanin, EGFR, p16 methylation, DNA aneuploidy (pooled CIs NR in extracted text)	—	No (single-marker pooled estimates)	CSC markers (CD44, ALDH1, BMI1) did not reach top 4 pooled biomarkers, possibly reflecting too few comparable longitudinal studies rather than lack of effect
**[3]**	Swain et al., 2014	No new quantitative data; synthesises mechanistic rationale (cytoskeletal remodelling, EMT)	—	—	No reproducible search strategy or quality appraisal; risk of double-counting primary studies it cites
**[4]**	Saluja et al., 2019	Overall CSC marker RR 3.31 (95% CI 2.72–4.02); CD133 RR 4.20 (2.55–6.92); ABCG2 RR 3.64 (2.12–6.25); podoplanin RR 3.45 (2.37–5.04)	Not applicable (fixed-effect pooled RR with low heterogeneity)	No (marker-specific pooled estimates)	Draws on a still-limited, partly overlapping primary literature (shared with this review's own included list)
[5]	Habiba et al., 2017	Podoplanin HR 2.62 (95% CI 1.04–7.59, p = 0.039); ALDH1 HR 3.02 (95% CI 1.39–7.38, p = 0.004); co-expression HR 3.64 (95% CI 1.71–8.25, $p < 0.001$)	Histological grade and co-marker expression in multivariable Cox models	Yes (podoplanin + ALDH1 co-expression)	Single-centre retrospective cohort; short mean follow-up (3.4 years), high event fraction, and no internal or external validation.
**[8]**	Monteiro et al., 2024	Pooled HR 3.72 (95% CI 2.40–5.76, P <0.00001), I2 = 0% (no heterogeneity)	Not applicable (pooled multivariable-adjusted HRs from primary studies)	No (podoplanin-specific pooled estimate)	Only six pooled cohorts; $I^2 = 0\%$ has low power to exclude heterogeneity and does not remove differences in scoring, thresholds, or populations; several cohorts are also evaluated individually here [5,15–19].
**[9]**	Briggs et al., 2025	Pooled HR 4.4 (95% CI 2.6–7.4); sensitivity 78.9% (69.7–86.2%), specificity 55.0% (49.4–60.6%), PPV 36.4% (32.9–40.2%), NPV 88.8% (84.4–92.1%)	Not applicable (meta-analytic pooling)	No (podoplanin alone; diagnostic-accuracy profile)	Substantial overlap with primary cohort studies already appraised individually in this review; low PPV/specificity temper HR/NPV

Ref.	Study	Principal Finding (95% CI Where Reported)	Adjustment Variables	Combined or Multi-Marker Model	Principal Methodological Limitation
**[10]**	Monteiro et al., 2021	Overall transformation rate 27.6% (1047/3783); results presented by marker with individual study hazard ratios/odds ratios; no single pooled effect size computed	Not applicable (narrative synthesis across 46 heterogeneous studies)	No (narrative synthesis of individual marker findings)	Search ended in February 2020; no pooled effect estimate was calculated, and the primary-study evidence overlaps with later linked reviews and with studies included separately in the present review.
**[11]**	Monteiro et al., 2025	9/14 studies reported multivariate analysis; Bmi-1, Tipe-2, copy number alteration, and DcR2 among markers retaining independent significance in those 9 studies; no single pooled HR/CI reported	Not applicable (varied by underlying primary study)	No single combined model (narrative synthesis across 14 heterogeneous studies)	Only 9 of 14 studies reported multivariable-adjusted findings; substantial overlap with the earlier linked review and with primary studies included separately.
**[12]**	Cousins & Carney, 2022	Restates source review's overall transformation rate 27.6% (3783 patients, 49 biomarkers); no new CI generated	—	—	Not independent evidence; restates and interprets Monteiro et al. [10] without a new search or original data.
**[13]**	Logeswari et al., 2014	Positivity 0% normal, 59.4% leukoplakia, 82.0% OSCC ($p < 0.001$); no CI reported	None (unadjusted proportions)	No	Cross-sectional, no malignant-transformation follow-up; antibody/scoring details not reported
**[14]**	Gissi et al., 2018	Podoplanin specificity 96.87% for dysplasia; multiple logistic regression chi-square 9.77 ($p < 0.01$); no CI reported	p53 and Ki-67 in the same multivariable logistic regression	Yes (podoplanin + p53 + Ki-67 multivariable model)	Very small sample (n = 8 dysplastic); no malignant-transformation follow-up
**[15]**	Kawaguchi et al., 2008	HR 3.09 (95% CI 1.53–6.23), p = 0.002, multivariate Cox regression	Histology (dysplasia) as cofactor	Yes (podoplanin + histology: 3% versus 38% 3-year cancer incidence)	Single-institution chemoprevention-trial cohort; may not represent general OPL population
**[16]**	de Vicente et al., 2013	HR 8.738 (95% CI 1.834–41.634), p = 0.007, multivariable survival analysis	Multivariate survival analysis performed; specific covariates NR	No (podoplanin alone)	Modest sample (n = 58) and 13 transformations; wide CI; single-centre retrospective cohort.
**[17]**	Monteiro et al., 2022	Podoplanin HR 10.148 (95% CI 1.503–68.532, p = 0.017); podoplanin+dysplasia grade HR 10.238 (95% CI 2.06–50.889, p = 0.004)	Univariate KM/log-rank screening then multivariate Cox regression	Yes (podoplanin + dysplasia grade)	Modest sample (52 OL, only 11 high-risk), wide CIs; own scoring system not externally validated
**[18]**	Kreppel et al., 2012	Univariate p = 0.003 (transformation), P = 0.009 (SIN grade); 5-year OCFS 100% (negative) versus 41.7% (highest expression); lost significance after multivariate adjustment (no HR/CI reported)	SIN grade in multivariate model	Attempted (podoplanin + SIN grade); neither retained significance	Modest sample (n = 60) likely underpowered for multivariate modelling

Ref.	Study	Principal Finding (95% CI Where Reported)	Adjustment Variables	Combined or Multi-Marker Model	Principal Methodological Limitation
**[19]**	Grochau et al., 2019	Significant association with SIN grade (p = 0.033) and age (p = 0.003); no HR/CI (no survival model)	None (chi-square univariate)	No	Cross-sectional grade-correlation focus, not an updated malignant-transformation/survival analysis with hazard ratios
**[20]**	Verma & Chandrashekar, 2019	Podoplanin significant with dysplasia grade (p = 0.009); NOT significant with malignant transformation (exact P NR); no CI reported	None (chi-square/Wilcoxon univariate)	No	Null result for the primary transformation outcome; modest event count (n = 15) limits power
**[21]**	Sundberg et al., 2021	Podoplanin: recurrent 29.0% versus non-recurrent 14.0% (not significant); combined p53+p63 HR 2.48 (95% CI 1.13–5.44, p = 0.024)	Multivariable Cox regression (covariates not reported in sufficient detail)	Yes (combined p53+p63 model); podoplanin itself not in the significant combined model	Podoplanin scored visually (not image-analysis) unlike other markers; outcome (recurrence) is a step removed from malignant transformation
**[22]**	Alkan et al., 2022	No statistically significant IHC differences between the four lesion groups despite markedly different clinical transformation rates; no CI (null result)	None reported	No	Null IHC finding despite dramatically different clinical behaviour across lesion subtypes
**[23]**	D'Souza et al., 2018	No significant difference in raw positivity between groups (HOL 100%, NHOL 93.3%, controls 80%); significant difference in podoplanin score between HOL/NHOL and with grade (exact P NR); no CI reported	None	No	Very small sample (n = 15/group); no malignant-transformation outcome
**[24]**	Shabir et al., 2024	Significant association with grade within dysplasia group (p = 0.028); not significant within benign or OSCC groups (p > 0.05); no CI reported	None reported	No	Modest sample, especially dysplasia subgroup (n = 20); cross-sectional, no transformation follow-up
**[25]**	Zhang et al., 2017	All 10 markers significant univariately (incl. podoplanin); final multivariable model retained P53 and CA9 (not podoplanin) + age + dysplasia grade	Age and dysplasia grade combined with markers in final nomogram	Yes (10-biomarker + clinical-variable nomogram; final model = P53+CA9+age+grade)	Podoplanin significant univariately but not retained in final multivariable model; single-institution (Korean) cohort
**[26]**	Saintigny et al., 2009	DeltaNp63 HR 3.308 (95% CI 1.663–6.580, p = 0.0007); combined DeltaNp63+podoplanin+EIC HR 4.372 (95% CI 1.912–9.992, p = 0.0005), 61% versus 15% 5-year incidence	Age, treatment, histology as cofactors	Yes (three-marker combined model)	Same single-institution chemoprevention-trial limitations as Kawaguchi cohort; DeltaNp63 not a core review marker

Ref.	Study	Principal Finding (95% CI Where Reported)	Adjustment Variables	Combined or Multi-Marker Model	Principal Methodological Limitation
**[27]**	Kuo et al., 1998	All ED/EH/NBM 100% positive for all 4 forms (no discrimination); CD44v7-8 fell to 35.5% in SCC versus 100% NBM ($p < 0.01$); loss of CD44v7-8 worse 5-year survival (68.5% versus 25.5%, $p = 0.0281$); no CI reported	None (univariate log-rank)	No	Very small premalignant groups (*n* = 10 each), ceiling effect; no follow-up for malignant transformation of the premalignant cohort
**[28]**	Dhumal et al., 2022	CD44 U-shaped pattern (100% normal, decreasing OPMD, 87.5% OSCC); ALDH1 significant across 3 groups ($p < 0.05$), higher in dysplastic versus non-dysplastic OPMD; low- versus high-risk dysplasia NOT significant ($p = 0.82$); no CI reported	None (unadjusted group comparisons)	No	Very small OPMD sample (*n* = 30, only 15 high-risk), likely underpowered for the low-/high-risk comparison
**[29]**	Surendran et al., 2017	CSC/niche marker expression increased with grade ($p < 0.05$); CD31high/CD44high enriched in high-risk group ($p < 0.05$); CXCR4high/SDF1high ($p = 0.04$); 18% of high-risk group co-expression positive; no CI reported	None (ANOVA/chi-square/t-test univariate)	Co-expression analysis (CD44+CD31+CXCR4+SDF1), not a formal regression model	Cross-sectional design, not a longitudinal transformation-risk cohort; post-hoc co-expression categories rather than a pre-specified prognostic model
**[30]**	Sunny et al., 2023	Sensitivity >83–90% depending on cohort/method; >88% sensitivity/specificity with ML integration; CIs NR in extracted text	Clinical parameters incorporated into ML model	Yes (CD44+SNA-1 cytology panel, further combined with clinical parameters via ML)	Diagnostic-classification/point-of-care development study, not a longitudinal transformation-risk cohort
**[31]**	Shah et al., 2018	CD44v6/CD44v10 significantly upregulated, SYNE1/miR34a significantly downregulated in OSCC saliva versus controls; leukoplakia showed lesser SYNE1 downregulation; no CI/exact P reported	None reported	No (individual marker comparisons; axis described mechanistically)	Very small leukoplakia subgroup (*n* = 10), used as comparator; no malignant-transformation follow-up
**[32]**	Choi et al., 2023	COL1A1-CD44 co-expression: GSE42743 r = 0.62 ($p = 3.54 \times 10^{-9}$); GSE65858 r = 0.68 (p = 9.58x10^-38); scRNA-seq epithelial cells r = 0.43 ($p = 7.47 \times 10^{-3}$); no HR/OR reported	Not applicable (correlation and functional analyses).	No formal combined clinical model (functional/mechanistic evidence)	Leukoplakia subgroup small (*n* = 4), embedded in broader multi-subsite HNSCC cohort; no conventional IHC scoring for CD44

Ref.	Study	Principal Finding (95% CI Where Reported)	Adjustment Variables	Combined or Multi-Marker Model	Principal Methodological Limitation
**[33]**	He et al., 2014	CD44 histoscore: normal 191.6 ± 18.3, OED 240.8 ± 20.4, OSCC 268.4 ± 13.5 (OSCC versus normal $p < 0.001$); CD163 associated with OS ($p = 0.0319$, $n = 38$); no HR/CI reported	None (ANOVA/Pearson correlation)	No	OED subgroup very small ($n = 6$–7), with no transformation follow-up; primary focus was CD68/CD163-positive tumour-associated macrophages, and CD44/CSC markers were secondary.
**[34]**	Momin et al., 2023	CD44 positivity 87.8% dysplasia, 83.3% OSCC; no CI, no formal *p*-value for grade correlation reported	None	No	Purely descriptive, cross-sectional; no malignant-transformation outcome; raw percentages do not clearly support a monotonic severity relationship
**[35]**	Onofrei et al., 2025	CD44 significantly increased across OPMD/OSCC versus normal ($p < 0.001$), highest in actinic cheilitis and OSCC; DEC1 relatively consistent across lesion types (no significant discriminative pattern); no CI reported	None	No (DEC1 and CD44 compared descriptively, not combined)	Cross-sectional, no transformation follow-up; small subgroup sizes (e.g., 18 OSCC cases)
**[36]**	Liu et al., 2012	ABCG2 OR 3.24 (95% CI 1.31–7.98, $p = 0.011$); BMI-1 OR 4.03 (95% CI 1.59–10.26, $p = 0.003$), multivariate logistic regression	Dysplasia grade and other clinicopathological variables	No (each marker's independent adjusted risk reported separately)	Single-centre retrospective design; variable follow-up analysed with logistic regression; possible overlap with related Shanghai reports [37–39].
**[37]**	Liu et al., 2013	ALDH1 HR 4.17 (95% CI 1.96–8.90, $p <0.001$); CD133 HR 2.86 (95% CI 1.48–5.55, $p = 0.002$)	Clinicopathological covariates; specific variables were not reported in sufficient detail.	No (independent adjusted estimates)	Single-institution retrospective cohort; patient independence from related Shanghai reports [36,38,39] could not be confirmed.
**[38]**	Sun et al., 2013	CD133 positivity: 0% normal, 29% non-progressing OLP, 80% progressing OLP, 100% OSCC ($p < 0.001$); OR 9.79 (95% CI 1.96–48.92, $p = 0.005$)	Variables beyond CD133 were not reported in sufficient detail for the logistic model.	No	Only 10 progression events and a wide CI; diagnostic uncertainty between OLP, OLL, and dysplastic lichenoid lesions; possible institutional overlap with [36,37,39].
**[39]**	Feng et al., 2020	ALDH1/Bmi1 expression significantly correlated with subsequent multiple/multifocal carcinomas; no HR/OR/CI reported	NR	No	Brief report with limited methodological detail and no effect estimate; possible patient overlap with Shanghai cohorts [36–38].

Ref.	Study	Principal Finding (95% CI Where Reported)	Adjustment Variables	Combined or Multi-Marker Model	Principal Methodological Limitation
**[40]**	Rao et al., 2020	All 3 markers increased significantly OED to OSCC ($p < 0.05$); ALDH1/OCT4 correlated with nodal metastasis; tri-marker-positive survival reduction (KM $p = 0.00$); no HR/CI reported	None reported (KM/log-rank univariate)	Yes (combined ALDH1+Bmi1+OCT4 tri-marker-positive analysis)	Short/variable follow-up (6–52 months); survival analysis centred on OSCC overall survival rather than OED-specific transformation-free survival
**[41]**	Tarle et al., 2022	ABCG2+nEGFR co-expression HR 12.84 (95% CI 2.15–76.44, $p = 0.005$); ABCG2 alone weaker independent predictor (HR/CI NR)	Covariates in the co-expression/interaction model were not reported in sufficient detail.	Yes (ABCG2 + nEGFR co-expression/interaction model)	Small sample ($n = 50$, 21 events), very wide CI (2.15–76.44); ABCG2 alone weaker than nEGFR
**[42]**	Klein et al., 2020	BMI-1/Ki-67 increased across normal-to-OSCC spectrum ($p < 0.05$); BMI-1 higher in OL versus normal ($p < 0.05$); Ki-67/BMI-1 correlation R = 0.26 ($p = 0.01$); no significant difference non-dysplastic versus dysplastic OL for BMI-1; no CI reported	None	No	Cross-sectional, no prospective follow-up; BMI-1 did not distinguish dysplastic from non-dysplastic leukoplakia
**[43]**	Matsubara et al., 2011	DeltaNp63-LI increased significantly with dysplasia severity ($p < 0.0001$); higher in transformed (49.3%) versus non-transformed (34.2%) OL, $p < 0.01$; OSCC 5-year OS 40.0% (high-LI) versus 76.1% (low-LI), $p < 0.05$; no CI/HR reported	None (no Cox regression/multivariate model for the OL transformation analysis)	No	Very small transformation event count (6/112, 5.4%); no multivariate/Cox regression or HR for OL transformation
**[44]**	Kiyosue et al., 2013	p75NTR-LI invariant across dysplasia severity in OL (no significant difference); in OSCC, p75NTR-LI increased with grade/invasion, poorer prognosis in high-LI group; no CI reported	None reported for OL group	No	Null finding for OL dysplasia grades; no malignant-transformation/follow-up data for the OL cohort at all
**[45]**	Abdulmajeed et al., 2013	ALDH1, CD24, CD44 significantly higher in OSCC versus normal/mild dysplasia ($p < 0.008$, Bonferroni-corrected); CD24 correctly discriminated 69% dysplastic versus normal, 71% OSCC in validation cohort; CD271 no significant differences; no CI reported	Bonferroni correction for multiple comparisons	No (each marker assessed individually)	Cross-sectional diagnostic-accuracy design, not longitudinal; CD24 could not distinguish mild versus severe dysplasia grades

Ref.	Study	Principal Finding (95% CI Where Reported)	Adjustment Variables	Combined or Multi-Marker Model	Principal Methodological Limitation
**[46]**	Wang et al., 2019	PIWIL2 positivity was 75.2% in OSCC; high expression was associated with shorter cancer-free survival (p = 0.018). In the oral leukoplakia cohort, PIWIL2 and dysplasia grade were independent predictors in multivariable Cox analysis; HR and 95% CI were not reported in sufficient detail.	Dysplasia grade (backward stepwise Cox regression)	No formal combined model (PIWIL2 and dysplasia grade each independently significant)	CD133/NANOG assessed as comparators, not independently powered predictors; attrition from 208 to 101 analysable cases
**[47]**	Miguel et al., 2023	High-risk ED more frequent in non-homogeneous leukoplakia (OR 7.66, 95% CI 1.43–41.04), tongue/floor-of-mouth location (OR 3.37, 95% CI 1.14–9.94), intense PDPN expression (OR 9.17, 95% CI 1.0–83.77); non-continuous PDPN more likely extensive E-cadherin loss (OR 5.81, 95% CI 1.18–28.55)	Logistic regression; the complete covariate set was not reported in sufficient detail.	No single combined risk model (individual OR estimates)	Cross-sectional (histological risk-grade outcome, not validated future-transformation prediction); wide CI for PDPN-HRED association, small high-risk subgroup (n = 23)
**[48]**	Tokozlu et al., 2024	OSCC significantly higher CD133 immunoreactivity and inflammation scores, significantly decreased E-cadherin, versus OED/OEH; Snail mRNA upregulated in 100% OSCC, 85% OED, 82.5% OEH (positive-expression subgroups); CI/p-values not fully specified	None reported	No	Snail upregulation nearly as common in OEH (82.5%) as OSCC (100%), limiting specificity; sample sizes/statistical details not fully specified
**[49]**	Vasudevan et al., 2026	'Interferon-inducible network and stemness induction' identified as key hub-gene networks in transformed lesions (MEIS1 among upregulated hub genes); T-reg/dendritic-cell enrichment differentiating dysplasia grades, T-helper enrichment specific to transformation; no HR/OR/CI (pathway-level statistics only)	Not applicable.	Not applicable (systems-biology network analysis rather than a clinical combined-marker model).	In-silico analysis of existing public datasets, not a new IHC-validated patient cohort; does not report protein-level expression of any specific named review marker
